# Projecting Impacts of Marine Heatwaves and Their Effects on Long‐Term Changes in Marine Ecosystems Over the 21st Century

**DOI:** 10.1111/gcb.71123

**Published:** 2026-09-27

**Authors:** Vianney Guibourd de Luzinais, William W. L. Cheung, Thomas Frölicher, Laurent Bopp, Gabriel Reygondeau, Didier Gascuel

**Affiliations:** ^1^ UMR Dynamics and Sustainability of Ecosystems: From Source to Sea (DECOD), Institut Agro, Ifremer INRAE Rennes France; ^2^ Institute for the Oceans and Fisheries The University of British Columbia Vancouver British Columbia Canada; ^3^ Department of Aquatic Resources (SLU Aqua) Swedish University of Agricultural Sciences Uppsala Sweden; ^4^ Climate and Environmental Physics University of Bern Bern Switzerland; ^5^ Oeschger Centre for Climate Change Research University of Bern Bern Switzerland; ^6^ Laboratoire de Météorologie Dynamique, LMD‐IPSL, École Normale Supérieure, Université PSL, École Polytechnique, Institut Polytechnique de Paris Sorbonne Université, CNRS Paris France; ^7^ Rosenstiel School of Marine, Atmospheric, and Earth Science University of Miami Miami Florida USA

## Abstract

Marine heatwaves (MHWs) are increasing in frequency, duration and intensity under climate change. Although current MHWs trigger broad ecological impacts, such as coral bleaching, mass mortality of seagrass meadows, and declines in important fish stocks, the cascading effects of MHWs on ecosystem structure and functions in the 21st century remain unclear. In this study, we utilise a global biomass flow modelling framework to analyse these impacts. Leveraging climate projections from two Earth system models (ESMs), we project marine food webs dynamics at a spatial resolution of 1° latitude and longitude for the global ocean under a high‐emission ‘no mitigation’ scenario, two MHW baseline definitions (fixed vs. moving baseline), and five organismal acclimation capacity scenarios to MHWs. We project that MHWs exacerbate declines in total consumer biomass under intensifying Global Warming Level (GWL), with spatial variations. Globally, under a scenario assuming a fixed baseline, MHWs' additional biomass decline increases with GWL at a rate ranging from 1.3% to 6.7% biomass loss per Celsius degree of warming, according to best‐to‐worst acclimation capacity scenarios to MHWs. Furthermore, the projected impacts of MHWs on marine biomass vary across the food web, with higher trophic levels being impacted by 0.8% more than lower trophic level compartments. Moreover, under a scenario that assumes a moving baseline, reflecting marine communities' capacity to adapt or acclimatise to climate change, the projected biomass loss from MHWs is reduced by an average of 3.7% per °C of global warming compared to fixed baseline simulations. We conclude that it is essential to incorporate extreme temperature events into climate change impact and risk assessments and examine the potential role of adaptive capacities of marine communities to inform robust climate responses and adaptations for biodiversity and fisheries. We emphasise that limiting global warming to below +2°C would significantly reduce MHWs' impacts.

## Graphical abstract

In this study, we used a global biomass flow modelling framework to quantify the specific impact of MHWs by comparing simulations with and without MHWs under different marine community resistance scenarios, focusing on *α* = 0.2. We analysed biomass changes relative to the 1995–2014 reference period and considered two baseline assumptions (fixed vs. moving). Our results show that using a moving baseline mitigates both the spatial extent and magnitude of MHW‐induced consumer biomass losses, highlighting the role of ecological resistance and acclimation capacity in modulating the effects of global warming and extreme events on marine food webs.

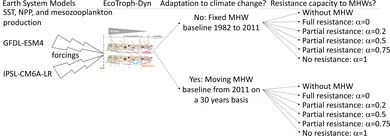

## 1 Introduction

Marine heatwaves (MHWs) are defined as periods of anomalously high sea surface temperatures that typically persist from days to months (Hobday et al. 2016; Oliver et al. 2018). Characterising future MHWs and assessing their biological impacts critically depend on the method used to establish the climatological baseline for defining MHWs. Using a fixed baseline period attributes all prolonged warming to anomalies and hence to MHWs (Frölicher et al. 2018; Oliver et al. 2021). Conversely, a moving baseline would attribute most of the prolonged warming to the long‐term trend in the climatology, effectively excluding most of them from MHWs (Capotondi et al. 2024; Jacox 2019; Oliver et al. 2021). Under the ‘no mitigation‐high emissions’ scenario (Shared Socioeconomic Pathway 5—Representative Concentration Pathway 8.5, or SSP5‐8.5), and using a fixed baseline defined by preindustrial or current climate conditions, MHWs are projected to strongly increase in intensity and frequency by the end of the 21st century, with the majority of ocean areas experiencing almost permanent heatwaves by 2070 (Frölicher et al. 2018; Guo et al. 2022; Oliver et al. 2019; Qiu et al. 2021), whereas the increase in MHWs days is reduced by around 1/3 and 2/3 under SSP2‐4.5 and SSP1‐2.6, respectively (Qiu et al. 2021). However, using a moving baseline definition of MHWs results in minor changes in MHW duration, intensity and frequency over the 21st century (Deser et al. 2024; Frölicher et al. 2018; Giménez et al. 2024; Jacox 2019).

There are varying perspectives on whether a baseline should remain constant or evolve in time (Amaya et al. 2023; Gupta 2023). A fixed baseline is more intuitive for understanding how species with limited adaptive capacity (proxy of low plasticity in their thermal niche breadth in response to climate change) encounter thermal fluctuations amidst warming trends (Oliver et al. 2021). In contrast, the moving baseline definition facilitates exploring how thermal fluctuations affect species with adaptive capabilities (proxy of plasticity in their thermal niche breadth in response to climate change), particularly during directional temperature changes such as those induced by contemporary global warming (Oliver et al. 2021). This adaptive baseline concept aligns with the hypothesis that communities within marine ecosystems exhibit varying capacities to adapt to future warming (Guo et al. 2022; Oliver et al. 2021).

Although the ability to adapt to long‐term warming is crucial, the resilience of marine ecosystems to MHWs depends on whether species and communities can withstand short periods of extreme temperature, during which they are undergoing thermal stress (most likely during the year's warmest months). Such events alter organisms' thermal environments, causing physiological stress and often mortality, unless mitigated by rapid physiological adjustments and elevated tolerance thresholds or vertical/spatial shifts (Morley et al. 2018; Seebacher et al. 2015). Under thermal stress, some species can rapidly initiate physiological responses to preserve their individual fitness (Guderley 1990; Seebacher et al. 2015). They prioritise essential survival processes, such as maintenance metabolism, whereas downregulating non‐essential maintenance such as anabolic metabolism (Donelson et al. 2018; Innis et al. 2021; Johansen et al. 2021; Munday et al. 2017). MHWs are expected to amplify the impacts of long‐term ocean warming at both individual and population levels, altering species interactions and triggering cascading effects throughout marine food webs that disrupt ecosystem structure and function (Arimitsu et al. 2021; Collins et al. 2019; Gomes et al. 2024; Harris et al. 2018; Pörtner and Roberts 2022). However, the specific contribution of MHWs to long‐term biomass loss and ecosystem disruption remains unquantified, particularly in relation to communities' adaptive capacity to climate change.

In this study, we used a trophodynamic approach to examine (1) whether MHWs would exacerbate the effects of long‐term impacts of ocean warming on marine ecosystem structure and functions and (2) how different levels of adaptive capacity of biological communities to temperature fluctuations would affect the impacts of MHWs on marine ecosystems in the 21st century. We especially analysed the effect of considering either a fixed (assuming no communities' adaptive capacity) or a moving (assuming communities' adaptive capacity) baseline for the definition of MHWs. We utilised the EcoTroph‐Dyn ecosystem model, to analyse the combined impacts of MHWs with gradual climate change over the 21st century on food web structure and functioning. EcoTroph‐Dyn has previously been applied to evaluate MHWs impacts on marine food web in the historical period (Guibourd de Luzinais et al. 2025). To focus on the MHWs most likely to affect marine communities, we specifically evaluated those occurring during the warmest month of the year, when organisms are likely to experience thermal stress. This study was conducted using simulations from two different Earth System Models (ESMs) to project ocean temperature, net primary production and mesozooplankton production (Tittensor et al. 2021), under the Shared Socioeconomic Pathway 5 and the Representative Concentration Pathway 8.5 (RCP‐SSP5‐8.5). We compared the results from five assumptions of community temperature sensitivities based on inferred resistance capacities to MHWs in the EcoTroph‐Dyn model. We hypothesised that (i) the additional biomass loss associated with MHWs' occurrence would exacerbate the impacts of global warming across ESMs and resistance capacity scenarios. (ii) Due to ESM inherent differences, the EcoTroph‐Dyn model would project, at an equivalent warming level, a different magnitude of MHW‐driven biomass change across the food web. (iii)Employing the alternative assumption of a ‘moving baseline’ in defining MHWs impacts would reduce the projected decrease in total consumer biomass in the 21st century. We first conducted a temporal analysis of MHWs' impact on biomass flow dynamics within the food web and its repercussions on the total consumer biomass at global scales using fixed and moving baselines. Subsequently, we investigated spatial pattern disparities between biomes and sensitivity to resistance capacity scenarios. Finally, we examined the diverse implications of future MHWs on food‐web trophic levels at the global and biomes scale.

## 2 Materials and Method

### 2.1 The EcoTroph Dynamic Model

#### 2.1.1 Biomass Flow and Fundamental Equations of EcoTroph‐Dyn

We utilised EcoTroph‐Dyn, a dynamic version of the EcoTroph trophodynamic modelling approach initially proposed by (Gascuel 2005) and later refined by Gascuel and Pauly (2009) and Gascuel et al. (2011). In EcoTroph, biomass is generated from primary producers (trophic level TL = 1) and consumed by heterotrophic organisms (TL > 1). This approach portrays food web dynamics as a continuous flow of biomass moving up the food web from primary producers (TL = 1) to top predators (high TLs, Figure 1). The resulting ecosystem structure is depicted as a continuous distribution of biomass across a range of TLs, known as biomass trophic spectra (Gascuel et al. 2005). Practically, the biomass trophic spectrum with TL above 1 is divided into small trophic classes defined by specific lower and upper trophic levels (with a conventional TL width of 0.1 in the steady‐state version of EcoTroph). The biomass spectrum in EcoTroph typically includes the entire biomass of consumers, encompassing organisms in pelagic, mid‐water, and benthic habitats. The time required for biomass to move from one trophic class to the next varies along the food chain, generally being faster at lower TLs compared to upper ones.

**FIGURE 1:**
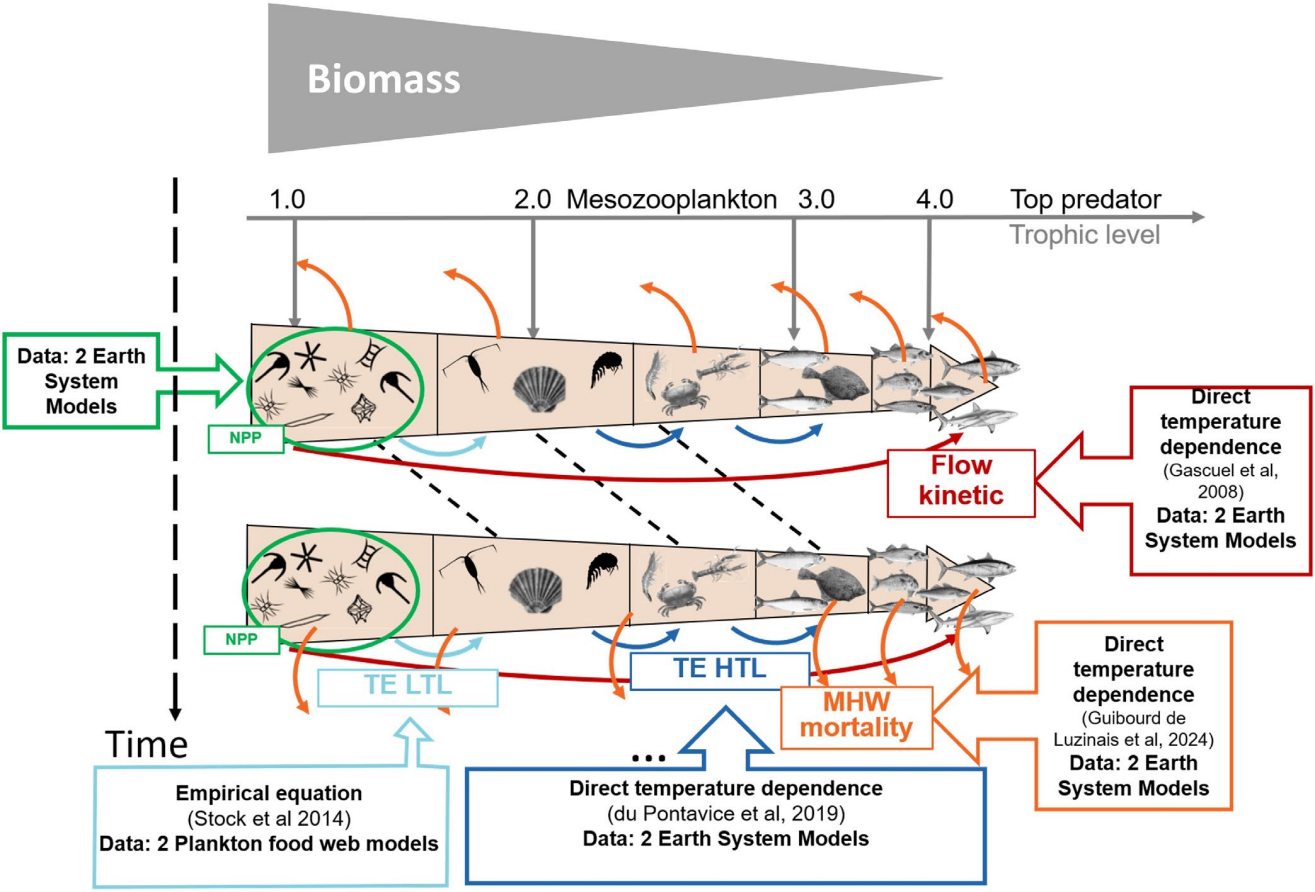
EcoTroph‐Dyn scheme (adapted from du Pontavice et al. 2021): The trophic functioning of marine food webs is represented by a biomass flow, starting with biomass entering the food web at trophic level 1 through net primary production (NPP). Biomass flow reaches each trophic level according to the two successive trophic transfer efficiencies. The flow kinetic (temperature‐ and MHW‐dependent) is crucial in defining trophic class boundaries and estimating biomass for each trophic level over time. When MHWs occur, there's an additional loss of biomass flow at each trophic level. The figure provides details on the empirical models used for each parameter: MHW loss rate, low trophic level transfer efficiency (TE LTL), high trophic level transfer efficiency (TE HTL) and flow kinetic.

From the EcoTroph steady‐state version, a dynamic version of the model called EcoTroph‐Dyn has been developed to assess the food web dynamic impacts of MHWs. EcoTroph‐Dyn simulates changes in biomass flows at a 15‐days time step to analyse how an MHW‐induced perturbation propagates over time through the modelled food web and quantify its cumulative impacts. The 15‐days time step was used as it closely represents the average duration of most naturally occurring or in vitro simulated MHWs (Smale et al. 2015); however, MHWs from 5 days duration are accounted (see Section 3.2, β coefficient). EcoTroph‐Dyn includes algorithms that model changes in flow kinetics boundaries of trophic classes and the resulting biomass states and flow between trophic classes, as well as an algorithm that models the biomass loss associated with MHWs' occurrence. EcoTroph‐Dyn algorithms' details are described in Appendix S1 and summarised here.

The biomass flow, defined as the quantity of biomass moving from one trophic class to the next due to predation or ontogeny, is not conservative and can be calculated at each time step as follows:

(1) $$\;{Ф}_{\tau +\Delta \tau ,t+1}={Ф}_{\tau ,t}\cdot e^{-\left(\mu_{t}+\eta_{\tau ,t}\right)\cdot {\Delta \tau }_{t}},$$

where τ corresponds to TL, $\mu_{\tau }$ (expressed in TL^−1^) represents the mean natural loss rate which defines the proportion of biomass flow that does not move up in the food web, due to non‐predation mortalities, excretion and respiration (du Pontavice et al. 2020; Gascuel et al. 2008). The $\eta_{\tau ,t}$ parameter (expressed in TL^−1^) represents the additional loss rate specifically due to mortalities induced by MHW events (Section 3.2).

The flow kinetic *K*_*τ*_ (expressed in TL·year^−1^), represents the velocity of biomass transfers from lower to higher trophic levels (TLs) (Gascuel et al. 2008) and is inversely proportional to biomass residence time (the duration each biomass unit remains at a given TL). To estimate *K*_*τ*_ we used the empirical equation proposed by Gascuel et al. (2008) which describes its variation as a function of SST and TL (τ):

(2) $$K_{\tau }=20.19\cdot \tau_{i}^{-3.258}\cdot e^{0.041\cdot {\mathrm{SST}}_{y}},$$

where SST_*y*_ corresponds to the annual moving average sea surface temperature in year *y*.

EcoTroph‐Dyn assumes that MHWs can increase the mortality of marine organisms and thus reduce their life expectancy. Consequently, according to equations used in the steady‐state version of EcoTroph, the flow kinetic during MHWs is increased as follows:

(3) $$\begin{array}{c} K_{\tau }=20.19\cdot \tau_{i}^{-3.258}\cdot e^{0.041\cdot {\mathrm{SST}}_{y}}\;\cdot \left(1+\eta_{\tau }\right), \end{array}$$

where $\eta_{\tau }$ is the MHW‐associated additional loss rate, only considered during MHW.

Biomass spectra in EcoTroph‐Dyn are divided into trophic classes with variable widths of trophic levels. ∆τ, the width of trophic classes [*τ*_*i*_; *τ*_*i*_ + 1[, is determined based on the estimated mean flow kinetics so that biomass could transfer up a trophic class in each time step (Δ*t* = 15 days). Thus:

(4) $$∆\tau =\tau_{i+1}-\tau_{i}=K_{\tau }\cdot \Delta t,$$

According to fluid dynamic equations, the EcoTroph‐Dyn state variable $\;B_{\tau ,t}$, representing the biomass present at time step *t* within the TL class [*τ*, *τ* + Δ*τ*[ is expressed as:

(5) $$B_{\tau ,t}=\frac{1}{K_{\tau ,t}}\cdot {Ф}_{\tau ,t}\cdot ∆\tau ,$$

where ${Ф}_{\tau ,t}$ is the mean quantity of biomass flowing within the trophic class [*τ*, *τ* + Δ*τ*[ at time step *t*, and $K_{\tau ,t}$ is the mean flow kinetic within the trophic class [*τ*, *τ* + Δ*τ*[.

Finally, the production *P*_*τ*,*t*_ of the trophic class [*τ*, *τ* + Δ*τ*[ at time step *t* is:

(6) $$P_{\tau ,t}=\int_{s=\tau }^{s=\tau +\Delta \tau }\Phi \left(s,t\right)\cdot \mathrm{ds}=\Phi_{\tau ,t}\cdot \Delta \tau ,$$

Hence, according to Equations (5) and (6), EcoTroph highlights that biomass stems from the ratio of production to flow kinetic. Production is expressed in *t*·TL·year^−1^, that is, biomass in weight moving up the food web by 1 TL on average during 1 year.

#### 2.1.2 Transfer Efficiency From Primary Production to Upper TLs

Equation (1) defines the transfer efficiency (TE) between continuous TLs, which corresponds to the estimated fraction of the biomass flow which is transferred from one TL to the next one (Eddy et al. 2021), as follows:

(7) $$\mathrm{TE}=e^{-\mu_{\tau }},$$

In this study, we modelled the trophodynamics of planktonic food webs separately from the upper part of the food web. We used projections of monthly average vertically integrated Net Primary Productivity (NPP) from 1950 to 2100 obtained from global coupled atmosphere–ocean‐biogeochemistry Earth System Models (ESMs). NPP is considered as biomass production at Trophic Level TL = 1, that is, *P*_1_ = NPP (Equation 5). We excluded consideration of detritus biomass flows. Although acknowledging the complexity of energy transfers within the planktonic food web (Friedland et al. 2012), previous analyses have consistently revealed a notable trend towards more efficient energy transfers to fish in highly productive regions (Armengol et al. 2019; Ryther 1969; Stock et al. 2014). This phenomenon is driven by two principal factors: the predominance of picophytoplankton in regions characterised by low productivity, resulting in extended food chains linking primary producers and fish, and the limited surplus energy available for small zooplankton in subtropical areas, leading to decreased planktonic transfer efficiency (Agawin et al. 2000; Heneghan et al. 2016; Stock et al. 2014; Stock and Dunne 2010).

To assess these variations in plankton food web transfer efficiency across the ocean, we used the estimated transfer efficiency between primary production and mesozooplankton production from du Pontavice et al. (2021), named TE LTL as:

(8a) $$\mathrm{TE}\;{\mathrm{LTL}}_{t,j}={\left(\frac{{\text{MEZOO PROD}}_{t,j}}{{\mathrm{NPP}}_{t,j}}\right)}^{1/\left(\text{MEZOO}\;{\mathrm{TL}}_{t,j}-1\right)},$$

where NPP_*t*,*j*_ is the net primary production provided by the considered ESM in the grid cell *j*, for the time step *t*. MEZOO PROD_*t*,*j*_ and MEZOO TL_*t*,*j*_ are the mesozooplankton production and trophic level in the grid cell *j* for the time step *t*. MEZOO TL_*t*,*j*_ has been calculated using biomass flows between mesozooplankton and its preys extracted from each ESM biogeochemistry compartment. These biogeochemistry compartment models are the Carbon, Ocean Biogeochemistry and Lower Trophics (COBALTv2) for Geophysical Fluid Dynamics Laboratory fourth generation ESM (GFDL‐ESM4; Stock et al. 2020) and the Pelagic Interaction Scheme for Carbon and Ecosystem Studies (PISCES2.0) for Institut Pierre‐Simon Laplace latest version ESM (IPSL; Aumont et al. 2015). Transfer efficiency of low TLs (TE LTL) was calculated each time step *t*, between 1950 and 2100 in every cell, *j*, of a two‐dimensional horizontal 1° × 1° grid covering the global ocean.

The transfer efficiency of low trophic levels (TLs), as defined in Equation (8a), is considered between TL = 1 and TL = 2. A linear transition is assumed between TL = 2 and TL = 3 from the transfer efficiency at low trophic levels (TE LTL), at TL = 2, to the transfer efficiency at high trophic levels (TE HTL), as elaborated in the following paragraph, at TL = 3 (du Pontavice et al. 2021).

For higher trophic levels (TL ≥ 3) the temperature‐dependent high TL transfer efficiencies (TE HTL) were estimated using the empirical equation established by du Pontavice et al. (2020):

(8b) $$\mathrm{TE}\;{\mathrm{HTL}}_{t,j}=e^{\left(-2.162+b\;+\left(-0.025+a\right)\cdot {\mathrm{SST}}_{t,j}\right)}\cdot 1.038013,$$

where *a* and *b* are specific parameters for each biome type (Appendix S2) and SST_*t*,*j*_ the sea surface temperature in cell *j* for the time step *t* simulated.

Previous EcoTroph applications modelled marine ecosystems on a yearly scale (du Pontavice et al. 2021, 2023), and changes in transfer efficiency reflected changes in species assemblages in response to long‐term ocean warming. In this study, transfer efficiencies are changing at a 15‐day time step. This higher‐frequency scale still captures long‐term shifts in species composition, but also allows us to assess how short‐term perturbations in energy flow and biomass during MHWs impact food‐web trophic transfer efficiency (Grimmelpont et al. 2023; Minuti et al. 2021).

### 2.2 MHW Loss Rate Algorithm Computation

We estimated MHW loss rate by combining sea surface temperature data with estimations of species biogeography and thermal niche. Given the strong positive correlation between NOAA AVHRR and ESM temperature datasets (Pearson's *r* = 0.85, Appendix S3), we reused the loss rate algorithm calibrated with NOAA AVHRR data established by Guibourd de Luzinais et al. (2025), whereas MHW detection over the 21st century (when to apply this loss rate algorithm) was performed directly with ESM time series (see Section 2.3.3). A detailed description of the MHW loss rate algorithm computation is provided in Appendix S4. Here, we explain the main steps for the incorporation of the effects of marine heatwaves in EcoTroph‐Dyn:

1. Detection and characterisation of MHWs from 1982 to 2021.
2. Estimation of species distribution and associated thermal niche based on (Reygondeau et al. 2026).
3. Matching MHWs' historical distributions and characteristics with species distribution.
4. Calculation of the percentage of species undergoing thermal stress in each ocean spatial cell.
5. Estimation, based on this percentage, of an additional loss rate algorithm associated with MHW as a function of their category and place of occurrence.

Finally, species were challenged by MHWs through the loss rate (*η*_*j*_) algorithm formulated following Guibourd de Luzinais et al. (2025), as follows:

(9) $$\eta_{j}=\exp^{-\exp^{b\_{\mathrm{tl}}_{j}\cdot \alpha \cdot \left({\mathrm{MHW}}_{\mathrm{cat}}-\frac{{\mathrm{lt}}_{50}\_{\mathrm{tl}}_{j}}{\alpha }\right)}}\;\cdot \beta ,$$

where b_tl_*j*_ and lt_50__tl_*j*_ correspond to the slope of the function and the index of marine heatwave intensity (MHW_cat,*j*_) at which 50% of the species are undergoing thermal stress in cell *j*, *β* corresponds to the MHW duration. MHW_cat_ corresponds to MHW intensity index (defined in Appendix S4; Hobday et al. 2018). *α* is a coefficient assumed to represent the species' resistance capacity to MHW conditions through reducing the mortality rate due to species' exposure to thermal stress. Although resistance capacity to MHWs is expected to vary among taxa because of their differences in biological and behavioural characteristics such as life history and mobility (e.g., Smith et al. 2023), here, we focus on the aggregated responses to MHWs across species and assume a fixed, community‐wide resistance parameter (α).

Resistance value of *α* = 0.2 was set as the baseline assumption for this study. This parameter was validated based on our previous work in which a setting α=0.2 resulted in relatively better representation of observed ecosystem responses and species' mortality rates during historical (1982–2021) MHW events when compared to alternative settings of α (Guibourd de Luzinais et al. 2025). Although α=0.2 represents a conservative and robust disturbance threshold, it reflects the severe, acute physiological stress observed over the world ocean during extreme thermal anomalies. In addition, we explored the sensitivity of the results to these species' resistance capacity to MHW conditions, testing four values of the coefficient *α*. These settings are full resistance (*α* = 0, that is, we fixed $\eta_{i}=0$; no mortality due to thermal stress), partial resistance (*α* = 0.2, 0.5; 20%, and 50% of the species die because of thermal stress, respectively) and no resistance (*α* = 1; all species die when they are under thermal stress). We also related the loss rate to the MHW duration within each 15‐day time step, assuming that heatwave duration increases the mortality rate (Kingsolver and Woods 2016; Rezende et al. 2014). The parameter β represents the fraction of the 15‐day time step during which an MHW occurs, ranging from β=0 (no MHW) to β=1 (MHW lasting the full 15 days of that step). Importantly, this parameter does not limit the total duration of an MHW to 15 days. Longer heatwaves are simulated across multiple consecutive time steps. For instance, a 60‐day heatwave would be modelled as four consecutive time steps, each with β=1, applying the corresponding 15‐day loss rate to each of those four periods.

### 2.3 EcoTroph‐Dyn Projections

#### 2.3.1 Fixed and Moving Baseline Simulations

We conducted two sets of projections employing fixed or moving baselines to investigate the ecological implications of species adaptation to global warming. In the first set, we employed a fixed baseline with MHWs detection and annual threshold defined relative to 1982–2011 conditions, representing no adaptation capacity to climate change. In the second set, we applied a moving baseline starting from 2011, representing adaptation capacity to climate change. Before 2012, the baseline remained consistent with set 1, whereas from 2012 onward, the baseline extended 30 years before the considered year. For instance, the baseline for 2012 encompassed the period 1983–2012; for 2013, it spanned from 1984 to 2013, and so forth into the future for projections.

#### 2.3.2 Application and Forcing Data

EcoTroph‐Dyn model has been applied separately to 32,389 grid cells in a spatial 1° × 1° grid covering the global ocean (Appendix S5). The biogeography of grid cells was delimited using the distribution of biomes identified by Reygondeau et al. (2013) and adapted from Longhurst (2007). Each cell was classified as one of the four biomes: tropical, temperate, polar or upwelling biomes. Biomass and production were calculated for TLs between 2.0 and 5.5. The EcoTroph‐Dyn model was forced by two models presenting contrasting projections of SST and NPP:GFDL‐ESM4 (Dunne et al. 2020; Stock et al. 2020) and IPSL‐CM6A‐LR (Boucher et al. 2020) SST and NPP output from 1950 to 2100 under the historical and SSP5‐8.5 scenario. Although Earth System Models (ESMs) provide subsurface temperatures, the use of SST maintains methodological consistency with established definitions of MHW (Hobday et al. 2016), and with projections of future MHWs also derived from SST. Moreover, a recent global analysis found that associations between annual temperature extremes and fisheries declines were robust across depth layers, with the use of bottom‐layer rather than surface temperature anomalies yielding consistent conclusions (Cheung et al. 2026).

Simulations testing, on a 15‐day time scale, indicated that a ‘burn‐in’ period of at least 10 years is needed for biomass spectra to reach equilibrium, whereas increasing the ‘burn‐in’ period beyond 12 years does not significantly change the equilibrium biomass spectra (*t*‐test, *p*‐value = 0.6118). Therefore, we used the averaged time series from 1900 to 1949 to pre‐run EcoTroph‐Dyn in each ocean spatial cell, over a burn‐in period of 12 years.

#### 2.3.3 Forcing Data Preparation and Scenarios

To assess extreme events' specific effects, we compare simulations with and without MHWs. We first identified MHW days from the ESMs' SST time series, using the SST data as‐is. We identified an MHW when the daily‐mean SSTs exceed an extreme temperature threshold value for at least five consecutive days (Hobday et al. 2016). The extreme temperature threshold value was calculated for each 1° latitude × 1° longitude spatial cell as the 90th percentile of daily‐mean SST from the 30‐year climatology (fixed or moving baseline). We did not calculate threshold values by season; thus, MHW events were identified by a single threshold across the year. As a result, we detected MHWs mostly occurring during the year's warmest months, corresponding to the period where species are more likely to undergo thermal stress. Although using a fixed annual threshold could penalise ‘normal’ summer warm events, employing a seasonally varying baseline would identify off‐season anomalies that do not exceed absolute thermal limits and thus lack direct, mortality‐inducing effects on the organisms. We used the R package heatwaveR described at https://robwschlegel.github.io/heatwaveR/ (Schlegel and Smit 2018) to detect MHWs and their characteristics (intensity and duration calculated from the threshold value) in each spatial cell from January 1950 to December 2100.

For the scenarios including MHWs, we directly used the SST time series from the ESMs (GFDL‐ESM4 and IPSL‐CM6A‐LR). In contrast, for the scenario excluding MHW, hereafter referred to as the ‘without MHW’ scenario, we applied a decomposition procedure to the daily SST time series to remove MHW signals. Specifically, we decomposed the daily SST time series (*Y*_*t*_) of each ocean spatial cell using a Census X‐11 procedure (Pezzulli et al. 2005; Shiskin 1967; Vantrepotte and Mélin 2011). With this method, the time series can be decomposed as follows:

(10) $$Y_{t}=T_{t}+S_{t}+H_{t},$$

With $Y_{t}$ the daily SST of day *t*, $T_{t}$ the underlying long‐term trend, $S_{t}$ the seasonal component (repetitive pattern over time), and $H_{t}$ an irregular component, which is the unexplained variation of the time series that is not attributed to trend or seasonality.

The $T_{t}$ underlying long‐term trend is obtained from the annual‐centred running average of the initial series $Y_{t}$. The $S_{t}$ the seasonal component, is then computed by applying a seasonal running mean to the trend‐adjusted series ($Y_{t}-T_{t}$) to derive seasonal coefficients avoiding any confusion with the inter‐annual (trend) signal. After revised estimates of these two components (see the method in Pezzulli et al. 2005; Vantrepotte and Mélin 2011), the residual component is computed as $\;H_{t}=Y_{t}-T_{t}-S_{t}$.

We applied the following procedure to create a daily SST time series in each cell for the scenarios without MHWs:

When the daily $Y_{t}$ value was tagged as an MHW day and $Y_{t}$ was above the value ($T_{t}+S_{t}$), we re‐assigned the value ($T_{t}+S_{t}$) to the initial daily SST. For all the other situations, that is, for MHW days with $Y_{t}$ below ($T_{t}+S_{t}$) or not an MHW day, we let the daily SST value $Y_{t}$ assigned to its original value (see Appendix S6, where the process of this time series is illustrated). This second time series will subsequently be referred to as ‘without MHW’. This MHW removal procedure was applied using the same method as the identification step; because the underlying long‐term trend $T_{t}$ is isolated during the Census X‐11 decomposition and left completely untouched during the replacement of extreme irregular spikes ($S_{t}$), the overarching climate change warming signal and mean summer increases are fully preserved in the ‘without MHW’ scenario. Finally, to match the daily SST time series to EcoTroph‐Dyn time resolution (15 days), we aggregated the SST of $Y_{t}$ and ‘without MHW’ time series at a 15‐day scale. Specifically, we averaged the daily SST for every 15‐day of a year. We then computed the proportion of MHWs days (*β*) within each 15‐day. Thus, *β* = 0 when no MHW day is identified in a 15‐day and *β* = 1 when MHW last for an entire 15‐day.

For Net Primary Production (NPP) and mesozooplankton production monthly data, the GFDL‐ESM4 (Dunne et al. 2020; Stock et al. 2020) and IPSL‐CM6A‐LR (Boucher et al. 2020) include some time series where NPP can be null, especially in ocean spatial cells within high latitude regions due to ice cover and whole nights in winter months. As EcoTroph cannot be initialized by null production at TL = 1, we withdrew from the analysis the ocean cells with such time series (8214 ocean spatial cells corresponding to 4.7% of the ocean surface), leaving 36,242 spatial cells (95.3% of the ocean surface) to run EcoTroph‐Dyn. Finally, to match the NPP and mesozooplankton production time series to EcoTroph‐Dyn time step (15 days), we duplicated the NPP and mesozooplankton production monthly values to cover both 15 days that constitute each month.

Simulations performed are summarised in Figure 2.

**FIGURE 2:**
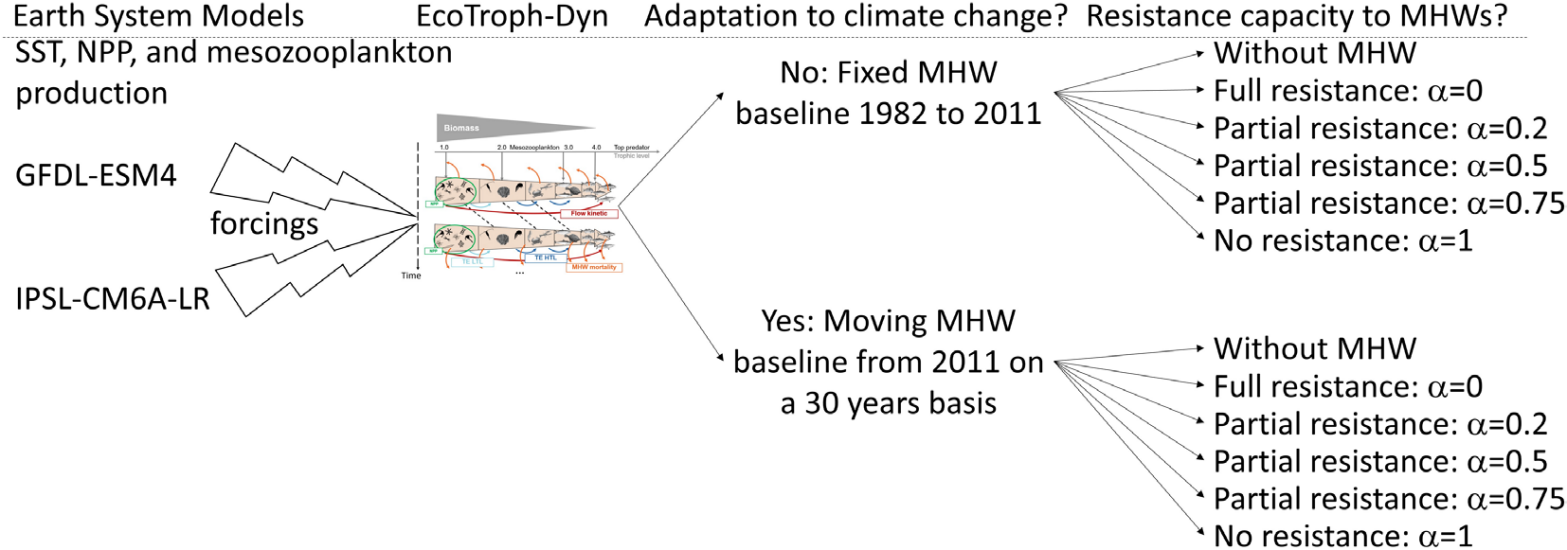
Schematic representation of the range of simulations. The first column shows the two ESMs accounted in the study and the respective environmental variables used to force the EcoTroph‐Dyn model. The third column describes the two assumptions of communities' capacities to adapt to long‐term climate change. The fourth column explains the different communities' resistance scenarios to MHWs.

From these simulations, we expressed the changes in the ecosystem biomass flow parameters and biomass at the global or local scale relative to the reference period 1995–2014. We computed the additional loss in biomass caused explicitly by MHWs as the difference between the biomass change values of the different resistance capacity simulations and the biomass change value of the ‘without MHW’ simulation. To evaluate the impact of MHWs over the 21st century, we analysed changes in ecosystem biomass as a function of global warming level (GWL) relative to the pre‐industrial period (1850–1900). Our main focus is on MHW impacts at a +2°C GWL. It represents the upper limit of the Paris Agreement's climate targets and serves as a critical benchmark widely utilised across the IPCC Sixth Assessment Report (AR6) and modern impact projection studies. In our simulations, this +2°C GWL corresponds to the year 2051 in the GFDL model and 2043 in the IPSL model.

## 3 Results

### 3.1 Extreme Temperature Events Impact Biomass Flow

The projected duration and mean intensity of MHWs increase with GWL levels and are consistent in trends between the IPSL and GFDL models (Figure 3a,b). For instance, in a +2°C global warming level relative to preindustrial levels (1850–1900) MHWs lasting around 120 days with an average temperature anomaly over the 90th threshold between 3.8°C and 4.5°C are expected under the fixed baseline approach (i.e., assuming no biological adaptation to climate change). However, under the moving baseline approach (i.e., assuming marine communities have adaptation capacities to climate change), most of the prolonged warming is attributed to the long‐term trend in the climatology, eliminating it from anomalies and MHWs, resulting in a much smaller increase in MHW duration and anomaly by nearly two‐thirds and one‐third, respectively, under a +2°C GWL. There was substantial spatial heterogeneity in the projected MHW characteristics. In tropical biomes, for instance, the number of MHW conditions under the +2°C GWL scenario was projected to have 112 days under GFDL and 128 days under IPSL according to the fixed baseline assumption (Appendix S7).

**FIGURE 3:**
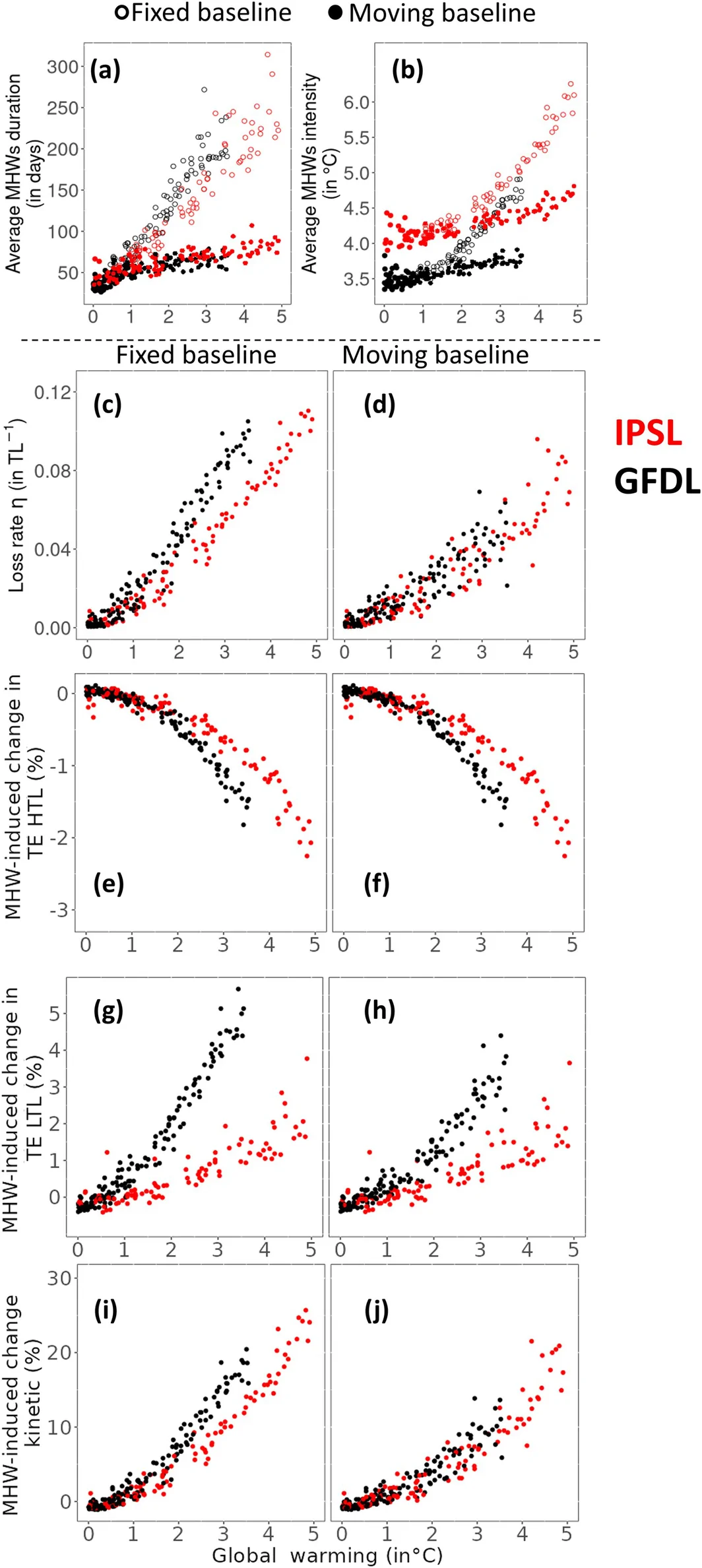
MHWs characteristics and impacts of MHWs on trophic spectra under different global warming (GWL). GWL is expressed relative to preindustrial levels (1850–1900). The EcoTroph‐Dyn simulations assumed a resistance capacity setting of *α* = 0.2. Each point in the scatterplots represents a yearly value. Panels (a, b) represent MHW duration and intensity, respectively. In panels (a, b), full and empty dots correspond to moving and fixed baselines, respectively. Panels (c, e, g, i) represent the additional changes induced by MHW (compared to the ‘without MHW’ scenario) in terms of loss rate, transfer efficiency at high and low TLs and flow kinetics, under the fixed baseline. Panels (d, f, h, j) represent the additional changes under the moving baseline. Red and black dots correspond to simulations using environmental forcings from IPSL and GFDL, respectively.

MHWs are expected to impose additional impacts on biomass flow dynamics. Specifically, under *α* = 0.2, the associated MHWs loss rate (*η*), which increases the celerity and decreases the quantity of biomass flowing into food webs, is expected to reach 0.04 under +2°C GWL and for the fixed baseline assumption in defining MHWs (Figure 3c). In contrast, this increase in loss rate would be mitigated by 39% under the assumption of communities being able to adapt to climate change (i.e., the moving baseline approach, Figure 3d). Similarly, the transfer efficiency, representing the proportion of biomass transferring across each trophic level, is expected to be impacted by MHWs. The transfer efficiency of the trophic level above 3 is expected to be negatively impacted by MHWs with an additional decrease around 0.5% compared to the ‘without MHW’ scenario under +2°C GWL (Figure 3e,f). However, the transfer efficiency of low trophic level groups is expected to be positively impacted by MHWs (Figure 3g,h). Notably, the estimated transfer efficiency is not dependent on the approaches in identifying MHWs (fixed and moving baseline assumptions) as transfer efficiency depends on the direct 15‐day ocean temperature, which is the same over both the fixed and moving baseline assumptions. Furthermore, the speed at which the biomass flows through the food webs is accelerated by MHWs occurrence and is increased quasi‐linearly with GWL (Figure 3i,j). This specific MHW increase in flow kinetic is expected to reach +5% under +2°C GWL, *α* = 0.2, fixed baseline assumption, irrespective of the Earth system model forcing. According to the different *α* scenarios, results strongly agree that biomass flow parameters change in response to MHWs; however, significant changes in magnitude are observed (ANOVA *p*‐value < 0.05). Finally, these temporal trends of biomass flow parameters exhibit important spatial discrepancies. Tropical, upwelling, and temperate biomes highlight greater changes than the Arctic and Antarctic biomes, resulting in different consequences on total consumer biomass (Appendix S8).

### 3.2 Total Consumer Biomass Responses to MHWs

MHWs are expected to exacerbate the declines in total consumer biomass under intensifying GWL. Under a resistance capacity assumption of *α* = 0.2, total consumer biomass is projected to decline by 16.0% ± 3.1% (standard deviation) and 7.0% ± 1.0% under 2°C GWL relative to preindustrial levels (1850–1900) for GFDL and IPSL, respectively (Figure 4a). Specifically, long‐term sea surface temperature trends (without MHWs) are projected to reduce total consumer biomass by 6.0% (GFDL) and 1.5% (IPSL) per Celsius degree of GWL. With MHWs, projected losses of consumer biomass were 2.1% (GFDL) and 2.2% (IPSL) of biomass more than without MHWs per Celsius degree of GWL (Figure 4b). Therefore, the projected total biomass losses, taking into account the MHW impact, would reach 8.1% and 3.7% per Celsius degree, in the two ESM, GFDL and IPSL, respectively. The consumer biomass loss driven specifically by MHWs, relative to the total impact of global warming (long‐term + MHW, i.e., with‐MHW scenario), increases with GWL (Figure 4c). At +2°C GWL, 25% and 52% of the total response to GWL (long‐term + MHW) would be due to the specific impact of MHWs for the GFDL and IPSL models, respectively.

**FIGURE 4:**
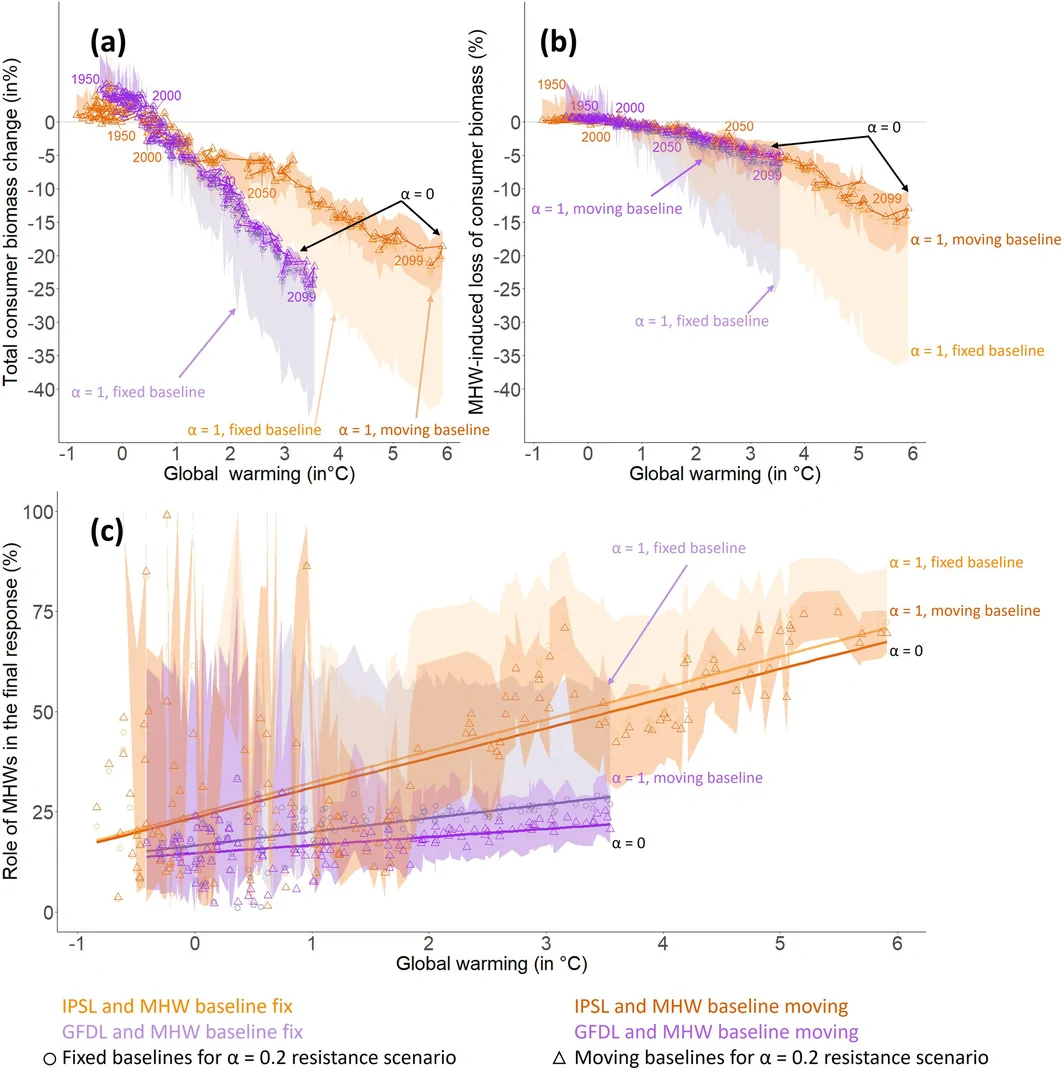
Projected changes in consumer biomass relative to global warming. (a) Change in total consumer biomass relative to the average across the reference period of 1995–2014, in the with‐MHW scenario, (b) loss of consumer biomass specifically associated with MHWs and (c) role of MHWs relative to change in consumer biomass due to global warming (i.e., with‐MHW scenario). The scenario of climate change is expressed as global warming level above the pre‐industrial levels. GFDL and IPSL forcings correspond to purple and orange colors respectively. Each marker represents a yearly value, with circle and triangle corresponding to fixed and moving baselines for *α* = 0.2 resistance scenario. Lines allow us to follow the temporal dynamic. Shade areas correspond to the response range between *α* = 0 (full resistance scenario) and *α* = 1 (all species die when they are under thermal stress). Light and dark shade areas correspond to the response range for the fixed and moving baselines, respectively. The horizontal grey line separates the positive and negative total consumer biomass changes.

The projected patterns of MHW impacts on total consumer biomass are robust to the tested parameters. More specifically, the rate of impacts is sensitive to the species' resistant capacity scenario to MHWs and to the MHWs baseline definition, but not the ESMs (Figure 4b). The moving baseline assumption reduces total consumer biomass loss compared to the fixed baseline, with an average attenuation of 3.7% ± 1.4% per °C of GWL across ESMs for *α* ≥ 0.5. Generalized Least Squares models (GLS) do not show any significant difference (ANOVA, *p*‐value > 0.05) between GFDL and IPSL at *α* resistance scenarios being equal. However, it is noteworthy that the combined effects of long‐term climate change and MHWs on total consumer biomass are more sensitive to different ESMs. The decrease in total consumer biomass is more pronounced under GFDL compared to IPSL, regardless of the resistance capacity assumption (Figure 4a) or the choice of fixed/moving baseline (light/dark shading). These different sensitivities of total consumer biomass can be mainly related to the trends in NPP and mesozooplankton biomass (EcoTroph‐Dyn forcing variables) in GFDL and IPSL models (Appendix S9). GFDL suggests a global maximum GWL of 3.5°C relative to the preindustrial levels (1850–1900) under SSP5‐8.5, accompanied by a 7.3% decrease in NPP and a 17.0% decrease in mesozooplankton biomass. In contrast, IPSL suggests a global maximum sea surface warming of 6.0°C, associated with an 8.6% increase in NPP and a 13.2% decrease in mesozooplankton biomass.

### 3.3 Spatial Differences in Projected MHW Impacts

Beyond this impact of MHWs at the scale of the global ocean, different responses from marine ecosystems are projected at the regional scale given the various characteristics of MHWs. Under GFDL, the specific total consumer biomass loss due to MHWs is important in lower latitudes, even with the more optimistic resistance scenarios of *α* = 0.2 (Figure 5a). Specifically, at +2°C GWL above preindustrial levels, under GFDL forcing, the decrease in consumer biomass, expected at low latitudes, is amplified by 35%–45% by MHWs under a fixed baseline (Figure 5a). This specific MHWs‐associated biomass decrease extends to higher latitudes with reduced resistance capacities scenario (Appendix S10, left column), regardless of the fixed or moving baseline assumption. The direction of the projected changes in total consumer biomass driven by MHWs is robust to different assumptions of resistance capacity to MHWs (Figure 5b), whereas the magnitude of the projected changes varies. Globally, the differences in the projected biomass loss between the no resistance and full resistance assumptions are greater than 20%, except in latitudes higher than 35° N and 55° S, where differences in the projected biomass loss are less than 20% additional biomass loss (Figure 5b). The small range of additional biomass loss in Indonesia's waters and the Philippines Sea corresponds to substantial additional biomass loss, exceeding 60%, due to MHWs regardless of the *α* assumption. Furthermore, the assumption that communities can adapt to climate change (i.e., moving baseline) does alleviate the additional effects of MHWs. Under the central hypothesis, *α* = 0.2 resistance scenarios, the moving baseline assumption leads to mitigation in the spatial extent of MHWs' effects with a reduction by a factor of 2 in the magnitude of the MHWs' additional total consumer biomass loss (Figure 5d). More generally, under this adaptive assumption, MHWs' effects on biomass are drastically reduced, as evidenced by the consistent, small range in MHW‐related biomass loss across various resistance scenarios to MHWs (Figure 5e and Appendix S10, right column). The dynamics of MHW characteristics can elucidate this consensus as a function of GWL upon baseline hypotheses (Figure 3a,b). Effectively, from +1°C GWL, MHW duration stagnates, and average intensity increases by less than 0.5°C under the moving baseline assumption. Consequently, this suggests a relatively small increase in MHWs impacts (additional biomass loss throughout the food web) over the 21st century.

**FIGURE 5:**
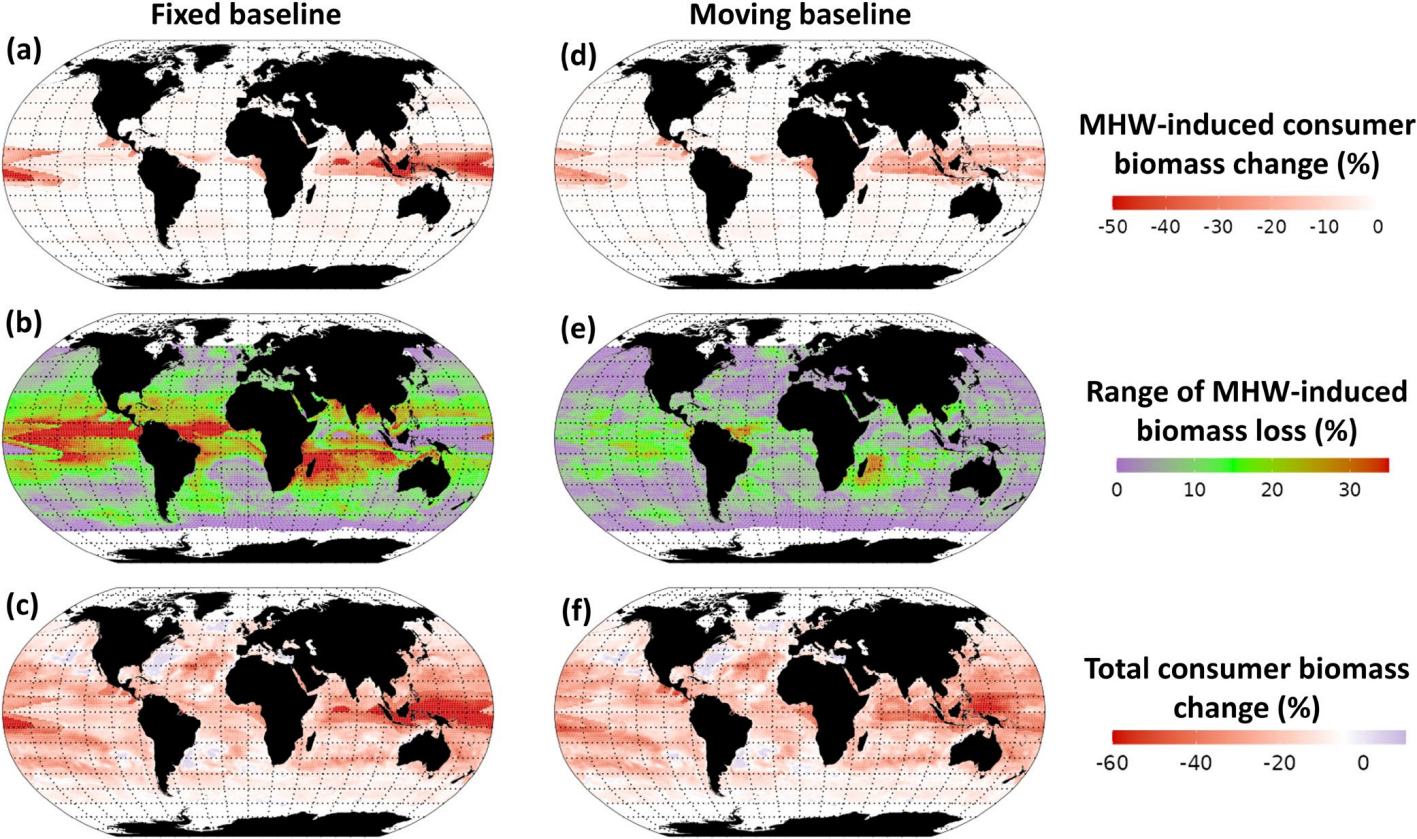
Spatial pattern of projected change in consumer biomass under a +2°C global warming level above preindustrial levels, under GFDL forcing. (a, d) Additional loss of consumer biomass associated with MHWs over the loss associated with long‐term warming under *α* = 0.2 scenario. (b, e) Range of additional loss of consumer biomass associated with MHWs according to *α* scenarios (from *α* = 0 to 1). (c, f) Spatial pattern of total consumer biomass change relative to the average across the reference period of 1995–2014 under *α* = 0.2 scenario. Panels (a–c) correspond to a fixed baseline, whereas panels (d–f) correspond to a moving baseline. Map lines delineate study areas and do not necessarily depict accepted national boundaries.

These spatial variabilities of MHWs' impacts contribute to exacerbating the effects of long‐term climate change. Together, under a 2°C GWL relative to pre‐industrial levels (in 2051 for GFDL Figure 5 and in 2043 for IPSL Appendix S11, respectively), with *α* = 0.2 and a fixed baseline assumption, total consumer biomass in most ocean areas is projected to decline (Figure 5c), particularly substantially in low latitudes, with an estimated biomass decrease between 50% and 60%. However, there are some exceptions like the area off the North American Pacific coast and of Central America. In higher latitudes, biomass losses are projected to be around 20%, except for the area off the Atlantic coast of Gibraltar Detroit where biomass is projected to decrease by nearly 40%. However, certain specific areas, such as the northern waters of Cuba, the South Atlantic Gyre, and the California Current, are exceptions to this general pattern of biomass decrease and are projected to gain up to 10% of total consumer biomass. The spatial patterns of projected changes in total consumer biomass are consistent with the trends in NPP and mesozooplankton biomass (Appendix S12). Specifically, positive total consumer biomass changes are expected only in areas with both positive NPP and mesozooplankton changes higher than 25%.

Analogous outcomes are projected under IPSL forcing, albeit with spatial variations and a higher magnitude in the response, reinforcing confidence in these results (Appendix S13).

### 3.4 Different Responses to MHWs Across Trophic Levels

On a global scale, in addition to long‐term climate change, MHWs exacerbate the differences in biomass losses between high trophic levels (TLs = 4–5.5) and low trophic levels (TL = 2–3) (Figure 6). Projections from the GFDL and IPSL without MHWs show that high TLs and low TLs lose 6.6% and 6.3% (GFDL) and 0.8% and 2.0% (IPSL) of biomass per degree Celsius of warming, respectively, resulting in a difference of +0.6% (GFDL) and −1.2% (IPSL). In GFDL and IPSL forcing scenarios with MHWs, a resistance *α* of 0.2, and a fixed baseline, high TLs and low TLs are expected to experience additional biomass losses of 2.6% and 2.1% per degree Celsius of GWL under GFDL, and 2.4% and 1.9% under IPSL, respectively (Figure 6a,b), resulting in an increased difference of +0.5% under both GFDL and IPSL. By increasing the value of *α* (decreasing the resistance capacity of communities to MHWs), the difference between projected changes in biomass under GFDL and IPSL further increases. Finally, the assumption of a mobile baseline shows an attenuation of the impacts of MHWs on trophic levels (Figure 6a,b), with smaller biomass losses for each trophic group. This reduction in the impact of MHWs is even more pronounced at the lower end of the food web due to the consequent reduction in loss rates associated with MHWs (*η*) compared to the assumption of a fixed baseline (Figure 6c,d). According to GFDL and with a resistance *α* of 0.2, the loss rate is halved between the assumption of a fixed and a mobile baseline for each trophic group.

**FIGURE 6:**
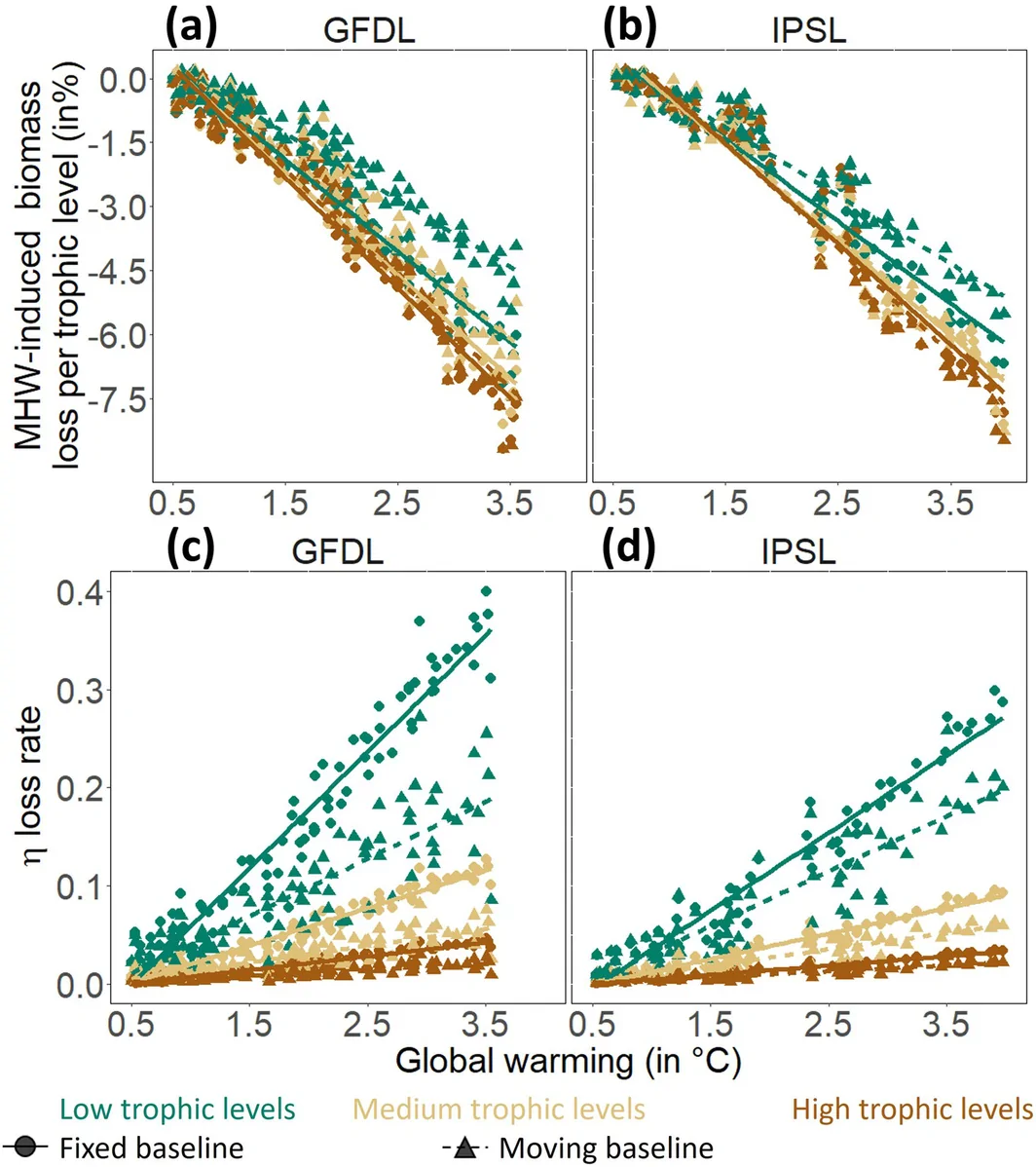
Projected impacts of MHWs per trophic group relative to global warming level according to GFDL and IPSL under the *α* = 0.2 scenario. (a, b) Additional loss of consumer biomass associated with MHWs, for GFDL (a) and IPSL (b). (c, d) Evolution of the loss rate *η*, for GFDL (c) and IPSL (d). Scenario of climate change is expressed as global warming above the pre‐industrial levels. Each marker represents a yearly value. Moving baseline corresponds to triangle and dashed lines, whereas circle and solid lines correspond to fixed baseline. Green, gold and brown correspond to low, medium, and high trophic levels, respectively.

Finally, projected differences in MHWs impacts on consumer biomass differ between biomes and trophic groups (Figure 7). Trophic groups of tropical biomes exhibit dramatic additional biomass loss compared to other biomes, reaching more than 10% of biomass loss for each trophic group under *α* = 0.2, GFDL and IPSL forcing, and fixed baseline. These biomass losses per trophic group decrease with reduced resistance capacity, yet they can be mitigated through the hypothesis of adaptation to climate change (moving baseline, see Appendix S13). The Tropical biome shows the largest difference in MHW‐related biomass loss between high (TL = 4–5.5) and low (TL = 2–3) trophic levels, with high TLs expecting to lose an additional 5% of biomass compared to low (Figure 7).

**FIGURE 7:**
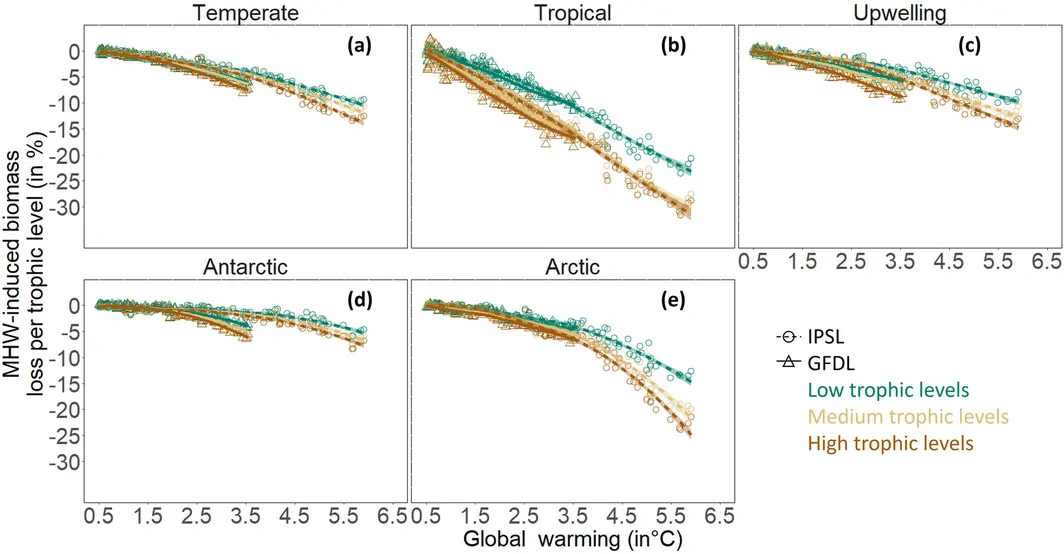
Projected changes in trophic group biomass per biome relative to the average across the reference period of 1995–2014 expressed as a function of global warming level above preindustrial levels according to GFDL and IPSL forcings, *α* = 0.2, and fixed baseline. Panels (a)–(e) correspond to the Temperate, Tropical, Upwelling, Antarctic, and Arctic biomes, respectively. Each marker represents a yearly value. Green, gold and brown correspond to low, medium and high trophic levels, respectively.

## 4 Discussion

In this study, we argue that MHWs are likely to exacerbate the long‐term impacts of climate change on marine ecosystems and the biomass they support by altering biomass flow (Collins et al. 2019). In addition, MHWs' exacerbation appears positively related to global warming levels. These projected impacts exhibit spatial variability due to the spatial dynamics of MHWs and differences in marine ecosystem functioning. The responses of marine ecosystems are consistent across different ESMs and resilience capacities of communities to MHWs (*α* values), with potential biomass losses being significantly mitigated under the assumption that species can adapt to climate change.

### 4.1 Large Impacts of Extreme Events

MHWs are anticipated to exert profound long‐term effects on the structure and functionality of marine ecosystems, potentially exacerbating the impacts of ocean warming. Projected increases in ocean temperatures are expected to amplify the duration, frequency, and intensity of MHWs (Frölicher et al. 2018; Oliver et al. 2021). These events are likely to influence biomass flow within ecosystems by altering the flow kinetics and transfer efficiency (Gascuel et al. 2008; Guibourd de Luzinais et al. 2024). MHWs also lead to substantial mortality of marine organisms, which is expected to reduce the flow of biomass through the food webs (Arimitsu et al. 2021; Gascuel et al. 2008; Gomes et al. 2024; Guibourd de Luzinais et al. 2024). Elevated water temperatures and MHWs are likely to favour short‐lived species with higher metabolic rates (Beukhof et al. 2019; du Pontavice et al. 2021, 2023). Increased dominance of short‐lived species has already been documented following MHW events, with a shift towards warmer‐water species and a reduction in colder‐water (long‐lived) species (Cavole et al. 2016; Smale et al. 2019). Consequently, MHWs are projected to accelerate and reduce the efficiency of biomass transfers by a factor of two to four compared to the long‐term increases in ocean temperature (depending on community resilience to MHWs).

Impacts of MHWs and long‐term changes of ecosystems are interrelated (Figure 4c), as revealed by the contrasts in the results driven by the two ESMs (Figure 4a). The GFDL and IPSL ESMs projected substantially different NPP, zooplankton biomass, and secondary producers for the 21st century, resulting in distinct changes in biomass flows predicted by EcoTroph‐Dyn. The IPSL model projected an increase in NPP and decrease in secondary producer biomass (Bopp et al. 2022), whereas the GFDL model projected a decrease in both (Kwiatkowski et al. 2020; Tittensor et al. 2021, Appendix S9). These global trends come with spatial variabilities (see Appendix S12). These differences in NPP and secondary producer biomass interact with temperature changes and affect the potential impacts of MHWs. Although IPSL is a ‘warm’ model that projects more intense MHWs (Figure 3b; Hausfather et al. 2022; Kwiatkowski et al. 2020), when positive NPP changes and mesozooplankton increases exceed 25%, these planktonic shifts can offset extreme temperature‐driven declines and foster localised ecosystem resilience. These specific conditions occurring less often under GFDL ESM severely enhance MHWs' biomass flow perturbations.

Despite these distinct underlying mechanics, both models project a consistent overall impact of MHWs on biomass fluxes and total consumer biomass, assuming equal community resilience (Figure 4b). However, the proportionate significance of MHWs differs between the two. Because the long‐term, background impacts of climate change are expected to be less severe under IPSL for a given global warming level (Guibourd de Luzinais et al. 2023; Tittensor et al. 2021), the acute disruptions caused by MHWs appear comparatively larger in the IPSL projections. Ultimately, as MHWs increase in duration and intensity throughout the 21st century, they will trigger stronger, more prolonged disturbances in biomass fluxes, causing overall MHW impacts to escalate alongside rising GWL (Figure 4c). Given the variability in the intensity, frequency, and duration of MHWs across ocean basins throughout the 21st century (Frölicher et al. 2018; Oliver et al. 2019), ecological consequences may be uneven across different oceans and biomes. Tropical regions, which are already more impacted by long‐term climate warming (du Pontavice et al. 2021; Tittensor et al. 2021), are also projected to experience more severe impacts from MHWs (Figure 5). In these ecosystems, when GWL exceeds +2°C relative to pre‐industrial levels, ocean surface temperatures are projected to exceed thresholds for most of the year (near‐permanent MHW) under the SSP5‐8.5 scenario, regardless of the ESM used (Oliver et al. 2019). Our projections estimated that biomass losses in tropical areas would be nearly two times more than in other biomes due to the large decrease in biomass flow. In contrast, despite the high intensity of projected MHWs in higher latitude biomes (temperate and upwelling areas), these biomes are expected to be ecologically less affected. Greater seasonality in these areas may provide species and communities with higher thermal safety margins, resulting in greater resistance to temperature changes (Nilsson et al. 2009; Pörtner and Farrell 2008; Pörtner and Peck 2010; Shultz et al. 2016). Consequently, less disruption of the biomass flow in these regions with smaller biomass loss is anticipated (Guibourd de Luzinais et al. 2024).

Our results also suggested that MHWs would exacerbate the amplification of biomass changes across TLs, increasingly from lower TLs to higher TLs, as impacts of climate change on ecosystem biomass and their flows propagated along the food web. The more pronounced decline in biomass at higher TLs results from the reduction in TE associated with increased temperature. This is consistent with the trophic amplification induced by long‐term increases in ocean temperature on a global scale (du Pontavice et al. 2021; Guibourd de Luzinais et al. 2023; Lotze et al. 2019). Finally, the intensification of MHWs combined with reduced resilience of marine communities (*α* value) would further exacerbate this amplification of biomass changes.

### 4.2 Biomass Loss Attenuation Associated With Moving Baselines Depends on Communities' Resistance to Extreme Temperature Events

In response to climate change and extreme temperature events, the structure and composition of marine communities depend on (1) their ability to adapt to long‐term ocean warming and (2) their capacity to withstand short‐term temperature fluctuations (Johansen et al. 2021). These two parameters are represented in our study by the application of a mobile baseline (Jacox 2019) and the use of different MHWs communities' resistance scenarios, respectively. If marine communities were able to fully adapt to long‐term climate change (moving baseline and increase of the threshold value used for MHWs detection over time), the impacts of MHWs would be lower compared to the no‐adaptation scenario (fixed baseline). Consequently, under the ‘moving baseline’ scenario, fewer days are classified as MHWs and the MHWs would be less intense (Figure 3a,b; Giménez et al. 2024). Consequently, the detected MHWs would lead to lower mortality rates (*η*, as the threshold increases with MHWs intensities/categories) and thus reduced disruptions in kinetic flow (Equation 3 and Figure 3j), resulting in substantial attenuation of biomass losses. However, biomass loss attenuation would be limited when communities have high resistance capacities (low *α* value, Figure 4) as in these *α* scenarios, communities do not suffer much from MHWs, whatever the assumption of adaptation to long‐term temperature fluctuation. For example, when resistance capacity is high (*α* = 0.2), MHW‐induced biomass losses would be attenuated by less than 10%, whereas with a low resistance capacity (*α* = 0.75 or 1), MHW‐induced biomass losses attenuation could reach up to 40%. However, while high resistance prevents immediate biomass collapse, it reduces natural selection pressure and can delay long‐term evolutionary adaptation (Ghalambor et al. 2007; Gilbert and Miles 2019). The rate of adaptation of marine communities to background ocean warming may not keep up with ocean warming, potentially limiting the applicability of the mobile baseline. The ‘Acclimation Response Ratio’ (ARR; Claussen 1977), defined as the change in upper thermal tolerance relative to the change in mean temperature, is generally less than 1. This means that a 1°C increase in environmental temperature does not correspond to a 1°C increase in thermal tolerance (Gunderson et al. 2016; Morley et al. 2019). Therefore, the full adaptation capacity of communities to long‐term climate change, enabling them to maintain ecosystem structure and function against rising temperatures would be unlikely and its likelihood would decrease with ocean warming. However, communities' resistance capacities (*α* values) could favour long‐term adaptation (Morley et al. 2019; Schaum and Collins 2014). This would be reflected in our modelling approach as a fixed baseline and low *α* value, which would gradually shift to a mobile baseline with ongoing ocean warming. Thus, the ‘true’ response of ecosystems would be in the middle, with a balance between a certain level of adaptation to long‐term changes and resilience to extreme events.

### 4.3 Uncertainties Related to the EcoTroph‐Dyn Model and Limitations

EcoTroph does not explicitly resolve the impacts of climate on individual species and populations but assumes that changes in environmental conditions will lead to the emergence of new biomass transfer characteristics. The dynamic version of EcoTroph (Guibourd de Luzinais et al. 2024) allows us to analyse the propagation of MHWs within food webs and their impacts over time. However, for future studies, it is important to discuss the limits and uncertainties of our results and explore model improvement possibilities.

Our representation of adaptive capacity relies on a moving MHW baseline, which serves as a proxy rather than a direct replication of biological processes (Giménez et al. 2024). In reality, evolutionary adaptation is a dynamic biological phenomenon driven by species‐specific traits, genetic diversity, and phenotypic plasticity (Foo and Byrne 2016; Sasaki and Dam 2019). By using a shifting mathematical baseline to represent these processes, our model assumes a generalised, predictable capacity for communities to adjust to temperature fluctuations. This proxy likely overlooks critical ecological nuances, such as varying rates of adaptation across trophic levels or threshold constraints (Hu et al. 2022; Verberk et al. 2026).

In this study, we focus on the high‐emission SSP5‐8.5 (RCP8.5) scenario, which could be a limitation as the late‐century tail of which is increasingly considered unlikely to occur. To mitigate this scenario‐dependent uncertainty and focus on actionable climate metrics, we frame our analysis on GWLs. This approach isolates the physical and ecological impacts at a shared GWL, independent of the exact timeline predicted by individual climate models. The scenario SSP5‐8.5 was selected because (i) its near‐term trajectory overlaps heavily with lower‐emission pathways and (ii) it encompasses the full range of GWLs within a single continuous run. We deliberately restrict our reported results to anomalies occurring at or below the +2°C GWL. By capping our analysis at this threshold, we ensure that our findings remain strictly within the plausible range of future climate forcing.

Although EcoTroph‐Dyn modelling framework is capable of capturing post‐disturbance recovery dynamics, as detailed in our previous work (Guibourd de Luzinais et al. 2024, 2025), the primary focus of this current study was to assess MHWs impacts over the 21st century on ecosystem biomass flow. In Guibourd de Luzinais et al. (2024), we have highlighted that the recovery times from one single MHW frequently exceed the return frequency of these extreme events. Consequently, even under optimistic resistance scenarios *α* = 0, successive MHWs interrupt ongoing ecosystem reconstruction. Given the projected rise in MHW frequency and intensity (Alexander et al. 2018; Frölicher et al. 2018; Oliver et al. 2019), ecosystems will increasingly lack the necessary window to fully rebuild before the next thermal shock occurs. Characterising these cumulative, multi‐decadal recovery dynamics remains critical for future research.

In our study, we accounted for variations in the structure of the planktonic food web through estimates of transfer efficiency at lower trophic levels. This integration is crucial for understanding biomass and production dynamics in marine ecosystems, as transfer efficiency at lower trophic levels limits the fraction of energy available for higher trophic levels (du Pontavice et al. 2021; Guibourd de Luzinais et al. 2024; Petrik et al. 2019). This consideration should provide more realistic estimates of the effects of climate change, although it does not capture the full diversity of pathways linking phytoplankton to fish. However, this is not sufficient, and future applications of EcoTroph‐Dyn should consider a more comprehensive approach to ecosystem functioning by integrating pelagic, benthic, and mesopelagic networks (du Pontavice et al. 2023; Petrik et al. 2019; Stock et al. 2017).

Additionally, while EcoTroph‐Dyn can incorporate top‐down effects induced by fishing pressures (e.g., du Pontavice et al. 2023; Gasche et al. 2012; Halouani et al. 2015), these are not included in our current model, which is governed solely by bottom‐up effects. Given that we have highlighted that higher TLs may be the most affected, the mitigation of predation pressure could lead to increased production of species at lower TLs (Gascuel et al. 2011). Consequently, we have likely underestimated the projected changes in the trophic structure of ecosystems.

The primary source of uncertainty in our projections of production and biomass stems from substantial inter‐model variability in NPP, zooplankton biomass, and secondary producers projections (Appendices S9 and S12; Kwiatkowski et al. 2020; Tittensor et al. 2021). In EcoTroph‐Dyn, as in other marine ecosystem models, primary production is a crucial component, as it supports and limits the biomass of higher trophic levels (e.g., Blanchard et al. 2012; Carozza et al. 2016; Cheung et al. 2011; Jennings and Collingridge 2015). Consequently, identifying current sources of uncertainty related to future net primary production and refining these estimates is one of the main challenges for understanding marine food web responses to climate change (Kwiatkowski et al. 2020). Moreover, a recent study (Sauterey et al. 2023) demonstrated that the assumption made by all ESMs—that phytoplankton communities are perfectly adapted to their thermal environment at all times—fails during MHWs. Thus, ESMs tend to overestimate primary production during MHWs throughout the 21st century. Given EcoTroph‐Dyn's strong reliance on NPP values, we may have significantly underestimated the impacts of MHWs on marine ecosystem functioning and biomass.

In response to MHWs, projected changes in the efficiency of biomass transfer at higher trophic levels and kinetic flows may result from shifts in species assemblages due to ocean warming (du Pontavice et al. 2020, 2021; Gascuel et al. 2008; Guibourd de Luzinais et al. 2024). However, other climate changes, such as ocean acidification, reduced dissolved oxygen, and the co‐occurrence of extreme events, can trigger additional biological responses that may alter the overall impacts of climate change on flow kinetics and trophic transfer efficiency (Gruber et al. 2021; Gunderson et al. 2016). The omission of these other climate components may render our approach conservative, suggesting that the projected decline in global marine biomass and production is likely underestimated.

Finally, we assumed that each grid cell represents an independent ecosystem with no exchange with adjacent cells. Within each oceanic cell, we presumed that SST, NPP and secondary production sustain the entire ecosystem without migration as a response to MHWs. This assumption is acceptable for benthic and demersal species, which are highly dependent on their habitats. However, it is more questionable for pelagic and mobile species, which might maintain their thermal niches by altering their distribution towards deeper waters or higher latitudes to retain their ecological traits (Cheung and Frölicher 2020; Crisp et al. 2009; Habary et al. 2017; Jacox et al. 2020; Lonhart et al. 2019). Such migrations would lead to a reorganisation of communities and trophic interactions within marine ecosystems, with the incorporation of species adapted to warmer waters (Gomes et al. 2024; Smith et al. 2023). Consequently, although these new species assemblages could alter marine ecosystem functions (Collins et al. 2019; Smale et al. 2019; Smale and Wernberg 2013; Smith et al. 2023), newly formed marine communities might be better equipped to cope with climate change and resist future MHWs.

### 4.4 Implications of Incorporating MHWs Into 21st‐Century Climate Change Projections

To discuss the importance of incorporating MHWs into projections of climate change impacts on marine ecosystems, we can compare our results with previous studies. These studies have focused solely on the long‐term effects of climate change in an ocean without fishing pressure on marine ecosystems (Guibourd de Luzinais et al. 2023; Tittensor et al. 2021). The strength of the Marine Ecosystems and Fisheries Model Intercomparison Project (Fish‐MIP; www.isimip.org/about/marine‐ecosystems‐fisheries/; Tittensor et al. 2018) lies in its integration of multiple ecosystem models based on different approaches, ranging from population‐based structures to functional trait and size‐based models. Our projections with the ‘full resistance’ scenario align with the steady‐state trends of EcoTroph (du Pontavice et al. 2021) and indicate a low or negligible additional impact of MHWs on marine ecosystems. This echoes the findings of Fredston et al. (2023), who showed no significant impact of past MHWs on global biomass catches. However, considering low resistance capacities (*α* = 0.5), the magnitude of the impacts of MHWs projected by EcoTroph‐Dyn could lead to a biomass loss up to twice as large as the long‐term climate change trends according to the stable version of EcoTroph (Appendix S14). This suggests that the projections from Fish‐MIP models, which are based solely on long‐term oceanic condition changes, may underestimate the total consumer biomass loss.

Finally, projections of the global impact of MHWs derived from other ecosystem models, such as those from the Fisheries and Marine Ecosystem Modelling Intercomparison Project (Fish‐MIP), would be extremely valuable. Given that these models use the same GFDL and IPSL Earth system models for their climate forcings, they already implicitly incorporate the same MHW events. However, most Fish‐MIP models address responses to long‐term ocean warming without explicitly isolating MHW events. Consequently, they could serve as an excellent ‘control’ against which to compare our approach. Furthermore, as we have already identified and isolated these MHW events within the forcing data, our event identification framework could be applied directly to these existing ecosystem models. Comparing results across such structurally diverse models will improve our understanding of inter‐model variability, refine our predictions and contribute to the improvement of management strategies for marine ecosystems facing extreme weather events.

## 5 Conclusion

This study highlights the profound impact that MHWs are expected to have on ecosystem structure and functioning by exacerbating changes in trophic transfer efficiency and biomass flow kinetics. These effects could lead to significant reductions in marine ecosystem biomass, with MHWs' impacts potentially representing more than 20% of biomass loss at a global scale, depending on species' resistance scenarios to extreme events. We also emphasise substantial spatial disparities, with projected impacts being much stronger in tropical ecosystems. Additionally, we emphasise a harmonised response through food webs, with stronger effects on higher trophic levels. It is crucial to note that the impacts of MHWs could be considerably mitigated by keeping global warming below +2°C compared to pre‐industrial levels. This underscores the critical importance of limiting greenhouse gas emissions.

## Author Contributions

**Vianney Guibourd de Luzinais:** conceptualization, investigation, writing – original draft, writing – review and editing, visualization, validation, methodology, formal analysis, software, data curation, resources. **William W. L. Cheung:** conceptualization, funding acquisition, methodology, validation, writing – review and editing, supervision, project administration, investigation. **Thomas Frölicher:** writing – review and editing, methodology, data curation. **Laurent Bopp:** data curation, methodology, writing – review and editing. **Gabriel Reygondeau:** writing – review and editing, data curation, methodology. **Didier Gascuel:** conceptualization, funding acquisition, writing – review and editing, methodology, validation, project administration, supervision, investigation.

## Funding

V.G.L. and D.G. acknowledge funding support from the Region Bretagne. W.W.L.C. and V.G.L. acknowledge funding support from the NSERC Discovery Grant and the SSHRC through the Solving‐FCB Partnership.

## Conflicts of Interest

The authors declare no conflicts of interest.

## Supporting information


**Appendix S1:** EcoTroph‐Dyn detailed.
**Appendix S2:** Transfer efficiency of higher trophic levels.
**Appendix S3:** ESM and NOAA AVHRR SST' time series.
**Appendix S4:** MHW loss rate algorithm computation.
**Appendix S5:** Study area.
**Appendix S6:** Time series decomposition.
**Appendix S7:** Number of MHWs days associated with a 2°C warming level above preindustrial levels (1850–1900).
**Appendix S8:** Biomes' biomass flow parameters responses to MHWs occurrence under global warming level above preindustrial level (1850–1900).
**Appendix S9:** NPP and mesozooplankton.
**Appendix S10:** Additional biomass loss associated with acclimation scenario to MHWs for both fixed and moving baseline.
**Appendix S11:** Maps of IPSL total consumer biomass change, standard deviation over acclimation scenario to MHWs, and MHWs‐associated additional biomass loss associated with a +2°C global sea surface warming level above preindustrial level.
**Appendix S12:** NPP and mesozooplankton map associated with a 2°C global sea surface warming level above preindustrial level (1850–1900) under GFDL and IPSL.
**Appendix S13:** Projected changes in trophic group biomass per biome relative to the average across the reference period of 1995–2014 expressed as a function of global warming level above preindustrial levels according to GFDL forcing, *α* = 0.2, and moving baseline.
**Appendix S14:** EcoTroph‐Dyn MHWs' simulation compared to Fish‐MIP models.

## Data Availability

Species occurrence data and associated trophic levels that support the findings of this study are openly available at https://doi.org/10.57745/PI0N92. The outputs of the two ESMs (GFDL‐EMS4 and IPSL‐CM6A‐LR) are publicly available as a part of the Coupled Model Intercomparison Project Phase 6 (CMIP6; https://pcmdi.llnl.gov/CMIP6/). Code availability: The code for the EcoTroph model can be found at https://doi.org/10.57745/NHVPCR.

## References

[gcb71123-bib-0001] Agawin, N. S. R. , C. M. Duarte , and S. Agustí . 2000. “Nutrient and Temperature Control of the Contribution of Picoplankton to Phytoplankton Biomass and Production.” Limnology and Oceanography 45, no. 3: 591–600. 10.4319/lo.2000.45.3.0591.

[gcb71123-bib-0002] Alexander, M. A. , J. D. Scott , K. D. Friedland , et al. 2018. “Projected Sea Surface Temperatures Over the 21st Century: Changes in the Mean, Variability and Extremes for Large Marine Ecosystem Regions of Northern Oceans.” Elementa: Science of the Anthropocene 6: 9. 10.1525/elementa.191.

[gcb71123-bib-0003] Amaya, D. J. , M. G. Jacox , M. R. Fewings , et al. 2023. “Marine Heatwaves Need Clear Definitions So Coastal Communities Can Adapt.” Nature 616, no. 7955: 29–32. 10.1038/d41586-023-00924-2. 37012469

[gcb71123-bib-0004] Arimitsu, M. L. , J. F. Piatt , S. Hatch , et al. 2021. “Heatwave‐Induced Synchrony Within Forage Fish Portfolio Disrupts Energy Flow to Top Pelagic Predators.” Global Change Biology 27, no. 9: 1859–1878. 10.1111/gcb.15556. PMC8048560 33577102

[gcb71123-bib-0005] Armengol, L. , A. Calbet , G. Franchy , A. Rodríguez‐Santos , and S. Hernández‐León . 2019. “Planktonic Food Web Structure and Trophic Transfer Efficiency Along a Productivity Gradient in the Tropical and Subtropical Atlantic Ocean.” Scientific Reports 9, no. 1: 2044. 10.1038/s41598-019-38507-9. PMC6376012 30765793

[gcb71123-bib-0006] Aumont, O. , C. Ethé , A. Tagliabue , L. Bopp , and M. Gehlen . 2015. “PISCES‐v2: An Ocean Biogeochemical Model for Carbon and Ecosystem Studies.” Geoscientific Model Development 8, no. 8: 2465–2513. 10.5194/gmd-8-2465-2015.

[gcb71123-bib-0007] Beukhof, E. , T. Dencker , L. Pecuchet , and M. Lindegren . 2019. “Spatio‐Temporal Variation in Marine Fish Traits Reveals Community‐Wide Responses to Environmental Change.” Marine Ecology Progress Series 610: 205–222. 10.3354/meps12826.

[gcb71123-bib-0008] Blanchard, J. L. , S. Jennings , R. Holmes , et al. 2012. “Potential Consequences of Climate Change for Primary Production and Fish Production in Large Marine Ecosystems.” Philosophical Transactions of the Royal Society, B: Biological Sciences 367, no. 1605: 2979–2989. 10.1098/rstb.2012.0231. PMC3479740 23007086

[gcb71123-bib-0009] Bopp, L. , O. Aumont , L. Kwiatkowski , et al. 2022. “Diazotrophy as a Key Driver of the Response of Marine Net Primary Productivity to Climate Change.” Biogeosciences 19, no. 17: 4267–4285. 10.5194/bg-19-4267-2022.

[gcb71123-bib-0010] Boucher, O. , J. Servonnat , A. L. Albright , et al. 2020. “Presentation and Evaluation of the IPSL‐CM6A‐LR Climate Model.” Journal of Advances in Modeling Earth Systems 12, no. 7: e2019MS002010. 10.1029/2019MS002010.

[gcb71123-bib-0011] Capotondi, A. , R. R. Rodrigues , A. Sen Gupta , et al. 2024. “A Global Overview of Marine Heatwaves in a Changing Climate.” Communications Earth & Environment 5, no. 1: 701. 10.1038/s43247-024-01806-9.

[gcb71123-bib-0012] Carozza, D. A. , D. Bianchi , and E. D. Galbraith . 2016. “The Ecological Module of BOATS‐1.0: A Bioenergetically Constrained Model of Marine Upper Trophic Levels Suitable for Studies of Fisheries and Ocean Biogeochemistry.” Geoscientific Model Development 9, no. 4: 1545–1565. 10.5194/gmd-9-1545-2016.

[gcb71123-bib-0013] Cavole, L. , A. Demko , R. Diner , et al. 2016. “Biological Impacts of the 2013–2015 Warm‐Water Anomaly in the Northeast Pacific: Winners, Losers, and the Future.” Oceanography 29, no. 2: 273–285. 10.5670/oceanog.2016.32.

[gcb71123-bib-0014] Cheung, W. W. L. , J. Dunne , J. L. Sarmiento , and D. Pauly . 2011. “Integrating Ecophysiology and Plankton Dynamics Into Projected Maximum Fisheries Catch Potential Under Climate Change in the Northeast Atlantic.” ICES Journal of Marine Science 68, no. 6: 1008–1018. 10.1093/icesjms/fsr012.

[gcb71123-bib-0015] Cheung, W. W. L. , and T. L. Frölicher . 2020. “Marine Heatwaves Exacerbate Climate Change Impacts for Fisheries in the Northeast Pacific.” Scientific Reports 10, no. 1: 6678. 10.1038/s41598-020-63650-z. PMC7174322 32317685

[gcb71123-bib-0016] Cheung, W. W. L. , T. L. Frölicher , I. Morgante , and J. Palacios‐Abrantes . 2026. in press. “Large Fisheries Declines Linked to Compound and Extreme Climate Events.” Nature Communications 17, no. 1: 8897. 10.1038/s41467-026-77077-z. PMC13534641 42680769

[gcb71123-bib-0017] Claussen, D. L. 1977. “Thermal Acclimation in Ambystomatid Salamanders.” Comparative Biochemistry and Physiology. Part A, Physiology 58, no. 4: 333–340. 10.1016/0300-9629(77)90150-5.

[gcb71123-bib-0018] Collins, M. , M. Sutherland , L. Bouwer , et al. 2019. “SPM6 Extremes, Abrupt Changes and Managing Risks 68.” 10.1017/9781009157964.008.

[gcb71123-bib-0019] Crisp, M. D. , M. T. K. Arroyo , L. G. Cook , et al. 2009. “Phylogenetic Biome Conservatism on a Global Scale.” Nature 458, no. 7239: 754–756. 10.1038/nature07764. 19219025

[gcb71123-bib-0020] Deser, C. , A. S. Phillips , M. A. Alexander , et al. 2024. “Future Changes in the Intensity and Duration of Marine Heat and Cold Waves: Insights From Coupled Model Initial‐Condition Large Ensembles.” Journal of Climate 37, no. 6: 1877–1902. 10.1175/JCLI-D-23-0278.1.

[gcb71123-bib-0021] Donelson, J. M. , S. Salinas , P. L. Munday , and L. N. S. Shama . 2018. “Transgenerational Plasticity and Climate Change Experiments: Where Do We Go From Here?” Global Change Biology 24, no. 1: 13–34. 10.1111/gcb.13903. 29024256

[gcb71123-bib-0022] du Pontavice, H. , D. Gascuel , S. Kay , and W. Cheung . 2023. “Climate‐Induced Changes in Ocean Productivity and Food‐Web Functioning Are Projected to Markedly Affect European Fisheries Catch.” Marine Ecology Progress Series 713: 21–37. 10.3354/meps14328.

[gcb71123-bib-0023] du Pontavice, H. , D. Gascuel , G. Reygondeau , A. Maureaud , and W. W. L. Cheung . 2020. “Climate Change Undermines the Global Functioning of Marine Food Webs.” Global Change Biology 26, no. 3: 1306–1318. 10.1111/gcb.14944. 31802576

[gcb71123-bib-0024] du Pontavice, H. , D. Gascuel , G. Reygondeau , C. Stock , and W. W. L. Cheung . 2021. “Climate‐Induced Decrease in Biomass Flow in Marine Food Webs May Severely Affect Predators and Ecosystem Production.” Global Change Biology 27, no. 11: 2608–2622. 10.1111/gcb.15576. 33660891

[gcb71123-bib-0025] Dunne, J. P. , L. W. Horowitz , A. J. Adcroft , et al. 2020. “The GFDL Earth System Model Version 4.1 (GFDL‐ESM 4.1): Overall Coupled Model Description and Simulation Characteristics.” Journal of Advances in Modeling Earth Systems 12, no. 11: e2019MS002015. 10.1029/2019MS002015.

[gcb71123-bib-0026] Eddy, T. D. , J. R. Bernhardt , J. L. Blanchard , et al. 2021. “Energy Flow Through Marine Ecosystems: Confronting Transfer Efficiency.” Trends in Ecology & Evolution 36, no. 1: 76–86. 10.1016/j.tree.2020.09.006. 33097289

[gcb71123-bib-0027] Foo, S. A. , and M. Byrne . 2016. “Chapter Two—Acclimatization and Adaptive Capacity of Marine Species in a Changing Ocean.” In Advances in Marine Biology, edited by B. E. Curry , vol. 74, 69–116. Academic Press. 10.1016/bs.amb.2016.06.001. 27573050

[gcb71123-bib-0028] Fredston, A. L. , W. W. L. Cheung , T. L. Frölicher , et al. 2023. “Marine Heatwaves Are Not a Dominant Driver of Change in Demersal Fishes.” Nature 621, no. 7978: 324–329. 10.1038/s41586-023-06449-y. 37648851

[gcb71123-bib-0029] Friedland, K. D. , C. Stock , K. F. Drinkwater , et al. 2012. “Pathways Between Primary Production and Fisheries Yields of Large Marine Ecosystems.” PLoS One 7, no. 1: e28945. 10.1371/journal.pone.0028945. PMC3262787 22276100

[gcb71123-bib-0030] Frölicher, T. L. , E. M. Fischer , and N. Gruber . 2018. “Marine Heatwaves Under Global Warming.” Nature 560, no. 7718: 360–364. 10.1038/s41586-018-0383-9. 30111788

[gcb71123-bib-0031] Gasche, L. , D. Gascuel , L. Shannon , and Y.‐J. Shin . 2012. “Global Assessment of the Fishing Impacts on the Southern Benguela Ecosystem Using an EcoTroph Modelling Approach.” Journal of Marine Systems 90, no. 1: 1–12. 10.1016/j.jmarsys.2011.07.012.

[gcb71123-bib-0032] Gascuel, D. 2005. “The Trophic‐Level Based Model: A Theoretical Approach of Fishing Effects on Marine Ecosystems.” Ecological Modelling 189, no. 3–4: 315–332. 10.1016/j.ecolmodel.2005.03.019.

[gcb71123-bib-0033] Gascuel, D. , Y.‐M. Bozec , E. Chassot , A. Colomb , and M. Laurans . 2005. “The Trophic Spectrum: Theory and Application as an Ecosystem Indicator.” ICES Journal of Marine Science 62, no. 3: 443–452. 10.1016/j.icesjms.2004.12.013.

[gcb71123-bib-0034] Gascuel, D. , S. Guénette , and D. Pauly . 2011. “The Trophic‐Level‐Based Ecosystem Modelling Approach: Theoretical Overview and Practical Uses.” ICES Journal of Marine Science 68, no. 7: 1403–1416. 10.1093/icesjms/fsr062.

[gcb71123-bib-0035] Gascuel, D. , L. Morissette , M. L. D. Palomares , and V. Christensen . 2008. “Trophic Flow Kinetics in Marine Ecosystems: Toward a Theoretical Approach to Ecosystem Functioning.” Ecological Modelling 217, no. 1–2: 33–47. 10.1016/j.ecolmodel.2008.05.012.

[gcb71123-bib-0036] Gascuel, D. , and D. Pauly . 2009. “EcoTroph: Modelling Marine Ecosystem Functioning and Impact of Fishing.” Ecological Modelling 220, no. 21: 2885–2898. 10.1016/j.ecolmodel.2009.07.031.

[gcb71123-bib-0037] Ghalambor, C. K. , J. K. McKAY , S. P. Carroll , and D. N. Reznick . 2007. “Adaptive Versus Non‐Adaptive Phenotypic Plasticity and the Potential for Contemporary Adaptation in New Environments.” Functional Ecology 21, no. 3: 394–407. 10.1111/j.1365-2435.2007.01283.x.

[gcb71123-bib-0038] Gilbert, A. L. , and D. B. Miles . 2019. “Antagonistic Responses of Exposure to Sublethal Temperatures: Adaptive Phenotypic Plasticity Coincides With a Reduction in Organismal Performance.” American Naturalist 194, no. 3: 344–355. 10.1086/704208. 31553209

[gcb71123-bib-0039] Giménez, L. , M. Boersma , and K. H. Wiltshire . 2024. “A Multiple Baseline Approach for Marine Heatwaves.” Limnology and Oceanography 69, no. 3: 638–651. 10.1002/lno.12521.

[gcb71123-bib-0040] Gomes, D. G. E. , J. J. Ruzicka , L. G. Crozier , D. D. Huff , R. D. Brodeur , and J. D. Stewart . 2024. “Marine Heatwaves Disrupt Ecosystem Structure and Function via Altered Food Webs and Energy Flux.” Nature Communications 15, no. 1: 1988. 10.1038/s41467-024-46263-2. PMC10937662 38480718

[gcb71123-bib-0041] Grimmelpont, M. , T. Milinkovitch , E. Dubillot , and C. Lefrançois . 2023. “Individual Aerobic Performance and Anaerobic Compensation in a Temperate Fish During a Simulated Marine Heatwave.” Science of the Total Environment 863: 160844. 10.1016/j.scitotenv.2022.160844. 36528094

[gcb71123-bib-0042] Gruber, N. , P. W. Boyd , T. L. Frölicher , and M. Vogt . 2021. “Biogeochemical Extremes and Compound Events in the Ocean.” Nature 600, no. 7889: 395–407. 10.1038/s41586-021-03981-7. 34912083

[gcb71123-bib-0043] Guderley, H. 1990. “Functional Significance of Metabolic Responses to Thermal Acclimation in Fish Muscle.” American Journal of Physiology. Regulatory, Integrative and Comparative Physiology 259, no. 2: R245–R252. 10.1152/ajpregu.1990.259.2.R245. 2201217

[gcb71123-bib-0044] Guibourd de Luzinais, V. , W. W. L. Cheung , and D. Gascuel . 2025. “Marine Heatwaves Deeply Alter Marine Food Web Structure and Function.” Biogeosciences 22, no. 21: 6583–6606. 10.5194/bg-22-6583-2025.

[gcb71123-bib-0045] Guibourd de Luzinais, V. , H. Du Pontavice , G. Reygondeau , et al. 2023. “Trophic Amplification: A Model Intercomparison of Climate Driven Changes in Marine Food Webs.” PLoS One 18, no. 8: e0287570. 10.1371/journal.pone.0287570. PMC10446190 37611010

[gcb71123-bib-0046] Guibourd de Luzinais, V. , D. Gascuel , G. Reygondeau , and W. W. L. Cheung . 2024. “Large Potential Impacts of Marine Heatwaves on Ecosystem Functioning.” Global Change Biology 30, no. 7: e17437. 10.1111/gcb.17437. 39054881

[gcb71123-bib-0047] Gunderson, A. R. , E. J. Armstrong , and J. H. Stillman . 2016. “Multiple Stressors in a Changing World: The Need for an Improved Perspective on Physiological Responses to the Dynamic Marine Environment.” Annual Review of Marine Science 8, no. 1: 357–378. 10.1146/annurev-marine-122414-033953. 26359817

[gcb71123-bib-0048] Guo, X. , Y. Gao , S. Zhang , et al. 2022. “Threat by Marine Heatwaves to Adaptive Large Marine Ecosystems in an Eddy‐Resolving Model.” Nature Climate Change 12, no. 2: 179–186. 10.1038/s41558-021-01266-5. PMC7612885 35757518

[gcb71123-bib-0049] Gupta, A. S. 2023. “Marine Heatwaves: Definition Duel Heats Up.” Nature 617, no. 7961: 465. 10.1038/d41586-023-01619-4. 37193814

[gcb71123-bib-0050] Habary, A. , J. L. Johansen , T. J. Nay , J. F. Steffensen , and J. L. Rummer . 2017. “Adapt, Move or Die – How Will Tropical Coral Reef Fishes Cope With Ocean Warming?” Global Change Biology 23, no. 2: 566–577. 10.1111/gcb.13488. 27593976

[gcb71123-bib-0051] Halouani, G. , D. Gascuel , T. Hattab , et al. 2015. “Fishing Impact in Mediterranean Ecosystems: An EcoTroph Modeling Approach.” Journal of Marine Systems 150: 22–33. 10.1016/j.jmarsys.2015.05.007.

[gcb71123-bib-0052] Harris, R. M. B. , L. J. Beaumont , T. R. Vance , et al. 2018. “Biological Responses to the Press and Pulse of Climate Trends and Extreme Events.” Nature Climate Change 8, no. 7: 579–587. 10.1038/s41558-018-0187-9.

[gcb71123-bib-0053] Hausfather, Z. , K. Marvel , G. A. Schmidt , J. W. Nielsen‐Gammon , and M. Zelinka . 2022. “Climate Simulations: Recognize the ‘Hot Model’ Problem.” Nature 605, no. 7908: 26–29. 10.1038/d41586-022-01192-2. 35508771

[gcb71123-bib-0054] Heneghan, R. F. , J. D. Everett , J. L. Blanchard , and A. J. Richardson . 2016. “Zooplankton Are Not Fish: Improving Zooplankton Realism in Size‐Spectrum Models Mediates Energy Transfer in Food Webs.” Frontiers in Marine Science 3: 201. 10.3389/fmars.2016.00201.

[gcb71123-bib-0055] Hobday, A. , L. V. Alexander , S. E. Perkins , et al. 2016. “A Hierarchical Approach to Defining Marine Heatwaves.” Progress in Oceanography 141: 227–238. 10.1016/j.pocean.2015.12.014.

[gcb71123-bib-0056] Hobday, A. J. , C. M. Spillman , J. P. Eveson , J. R. Hartog , X. Zhang , and S. Brodie . 2018. “A Framework for Combining Seasonal Forecasts and Climate Projections to Aid Risk Management for Fisheries and Aquaculture.” Frontiers in Marine Science 5: 137. 10.3389/fmars.2018.00137.

[gcb71123-bib-0057] Hu, N. , P. E. Bourdeau , C. Harlos , Y. Liu , and J. Hollander . 2022. “Meta‐Analysis Reveals Variance in Tolerance to Climate Change Across Marine Trophic Levels.” Science of the Total Environment 827: 154244. 10.1016/j.scitotenv.2022.154244. 35245550

[gcb71123-bib-0058] Innis, T. , L. Allen‐Waller , K. T. Brown , et al. 2021. “Marine Heatwaves Depress Metabolic Activity and Impair Cellular Acid–Base Homeostasis in Reef‐Building Corals Regardless of Bleaching Susceptibility.” Global Change Biology 27, no. 12: 2728–2743. 10.1111/gcb.15622. 33784420

[gcb71123-bib-0059] Jacox, M. G. 2019. “Marine Heatwaves in a Changing Climate.” Nature 571, no. 7766: 485–487. 10.1038/d41586-019-02196-1. 31332352

[gcb71123-bib-0060] Jacox, M. G. , M. A. Alexander , S. J. Bograd , and J. D. Scott . 2020. “Thermal Displacement by Marine Heatwaves.” Nature 584, no. 7819: 82–86. 10.1038/s41586-020-2534-z. 32760046

[gcb71123-bib-0061] Jennings, S. , and K. Collingridge . 2015. “Predicting Consumer Biomass, Size‐Structure, Production, Catch Potential, Responses to Fishing and Associated Uncertainties in the World's Marine Ecosystems.” PLoS One 10, no. 7: e0133794. 10.1371/journal.pone.0133794. PMC4520681 26226590

[gcb71123-bib-0062] Johansen, J. L. , L. E. Nadler , A. Habary , A. J. Bowden , and J. Rummer . 2021. “Thermal Acclimation of Tropical Coral Reef Fishes to Global Heat Waves.” eLife 10: e59162. 10.7554/eLife.59162. PMC7837695 33496262

[gcb71123-bib-0063] Kingsolver, J. G. , and H. A. Woods . 2016. “Beyond Thermal Performance Curves: Modeling Time‐Dependent Effects of Thermal Stress on Ectotherm Growth Rates.” American Naturalist 187, no. 3: 283–294. 10.1086/684786. 26913942

[gcb71123-bib-0064] Kwiatkowski, L. , O. Torres , L. Bopp , et al. 2020. “Twenty‐First Century Ocean Warming, Acidification, Deoxygenation, and Upper‐Ocean Nutrient and Primary Production Decline From CMIP6 Model Projections.” Biogeosciences 17, no. 13: 3439–3470. 10.5194/bg-17-3439-2020.

[gcb71123-bib-0105] Longhurst, A. R. , 2007. Ecological geography of the sea, 2nd Edn., 542. Elsevier. ISBN 978‐0‐12‐455521‐1.

[gcb71123-bib-0065] Lonhart, S. I. , R. Jeppesen , R. Beas‐Luna , J. A. Crooks , and J. Lorda . 2019. “Shifts in the Distribution and Abundance of Coastal Marine Species Along the Eastern Pacific Ocean During Marine Heatwaves From 2013 to 2018.” Marine Biodiversity Records 12, no. 1: 13. 10.1186/s41200-019-0171-8.

[gcb71123-bib-0066] Lotze, H. K. , D. P. Tittensor , A. Bryndum‐Buchholz , et al. 2019. “Global Ensemble Projections Reveal Trophic Amplification of Ocean Biomass Declines With Climate Change.” Proceedings of the National Academy of Sciences of the United States of America 116, no. 26: 12907–12912. 10.1073/pnas.1900194116. PMC6600926 31186360

[gcb71123-bib-0067] Minuti, J. J. , M. Byrne , D. A. Hemraj , and B. D. Russell . 2021. “Capacity of an Ecologically Key Urchin to Recover From Extreme Events: Physiological Impacts of Heatwaves and the Road to Recovery.” Science of the Total Environment 785: 147281. 10.1016/j.scitotenv.2021.147281. 33933766

[gcb71123-bib-0068] Morley, S. A. , L. S. Peck , J. Sunday , S. Heiser , and A. E. Bates . 2018. Acclimation Potential of Global Ectothermic Species, Collated From Literature, 1960 to 2015 (Version 1, p. 150 kB) [Application/vnd.ms‐excel]. Polar Data Centre, Natural Environment Research Council. 10.5285/20010BFB-C6D3-430F-B1F7-D16790AB8359.

[gcb71123-bib-0069] Morley, S. A. , L. S. Peck , J. M. Sunday , S. Heiser , and A. E. Bates . 2019. “Physiological Acclimation and Persistence of Ectothermic Species Under Extreme Heat Events.” Global Ecology and Biogeography 28, no. 7: 1018–1037. 10.1111/geb.12911.

[gcb71123-bib-0070] Munday, P. L. , J. M. Donelson , and J. A. Domingos . 2017. “Potential for Adaptation to Climate Change in a Coral Reef Fish.” Global Change Biology 23, no. 1: 307–317. 10.1111/gcb.13419. 27469983

[gcb71123-bib-0071] Nilsson, G. E. , N. Crawley , I. G. Lunde , and P. L. Munday . 2009. “Elevated Temperature Reduces the Respiratory Scope of Coral Reef Fishes.” Global Change Biology 15, no. 6: 1405–1412. 10.1111/j.1365-2486.2008.01767.x.

[gcb71123-bib-0072] Oliver, E. C. J. , J. A. Benthuysen , S. Darmaraki , et al. 2021. “Annual Review of Marine Science Marine Heatwaves.” Annual Review of Marine Science 13, no. 1: 313–342. 10.1146/annurev-marine-032720-095144. 32976730

[gcb71123-bib-0073] Oliver, E. C. J. , M. T. Burrows , M. G. Donat , et al. 2019. “Projected Marine Heatwaves in the 21st Century and the Potential for Ecological Impact.” Frontiers in Marine Science 6: 734. 10.3389/fmars.2019.00734.

[gcb71123-bib-0074] Oliver, E. C. J. , M. G. Donat , M. T. Burrows , et al. 2018. “Longer and More Frequent Marine Heatwaves Over the Past Century.” Nature Communications 9, no. 1: 1324. 10.1038/s41467-018-03732-9. PMC5893591 29636482

[gcb71123-bib-0075] Petrik, C. M. , C. A. Stock , K. H. Andersen , P. D. van Denderen , and J. R. Watson . 2019. “Bottom‐Up Drivers of Global Patterns of Demersal, Forage, and Pelagic Fishes.” Progress in Oceanography 176: 102124. 10.1016/j.pocean.2019.102124.

[gcb71123-bib-0076] Pezzulli, S. , D. B. Stephenson , and A. Hannachi . 2005. “The Variability of Seasonality.” Journal of Climate 18, no. 1: 71–88. 10.1175/JCLI-3256.1.

[gcb71123-bib-0077] Pörtner, H. O. , and A. P. Farrell . 2008. “Physiology and Climate Change.” Science 322, no. 5902: 690–692. 10.1126/science.1163156. 18974339

[gcb71123-bib-0078] Pörtner, H. O. , and M. A. Peck . 2010. “Climate Change Effects on Fishes and Fisheries: Towards a Cause‐and‐Effect Understanding.” Journal of Fish Biology 77, no. 8: 1745–1779. 10.1111/j.1095-8649.2010.02783.x. 21078088

[gcb71123-bib-0079] Pörtner, H.‐O. , and D. C. Roberts . 2022. “Climate Change 2022: Impacts, Adaptation and Vulnerability.”

[gcb71123-bib-0081] Qiu, Z. , F. Qiao , C. J. Jang , L. Zhang , and Z. Song . 2021. “Evaluation and Projection of Global Marine Heatwaves Based on CMIP6 Models.” Deep Sea Research Part II: Topical Studies in Oceanography 194: 104998. 10.1016/j.dsr2.2021.104998.

[gcb71123-bib-0082] Reygondeau, G. , Y. Egorova , K. Boerder , et al. 2026. “AquaX: An Enhanced and Revised AquaMaps Framework to Model Marine Species Distributions and Biodiversity.” PLoS One 21, no. 2: e0335823. 10.1371/journal.pone.0335823. PMC12923144 41719360

[gcb71123-bib-0104] Reygondeau, G. , A. Longhurst , E. Martinez , et al. 2013. “Dynamic biogeochemical provinces in the global ocean: DYNAMIC BIOGEOCHEMICAL PROVINCES.” Global Biogeochemical Cycles 27, no. 4: 1046–1058. 10.1002/gbc.20089.

[gcb71123-bib-0083] Rezende, E. L. , L. E. Castañeda , and M. Santos . 2014. “Tolerance Landscapes in Thermal Ecology.” Functional Ecology 28, no. 4: 799–809. 10.1111/1365-2435.12268.

[gcb71123-bib-0084] Ryther, J. H. 1969. “Photosynthesis and Fish Production in the Sea.” Science 166, no. 3901: 72–76. 10.1126/science.166.3901.72. 5817762

[gcb71123-bib-0085] Sasaki, M. C. , and H. G. Dam . 2019. “Integrating Patterns of Thermal Tolerance and Phenotypic Plasticity With Population Genetics to Improve Understanding of Vulnerability to Warming in a Widespread Copepod.” Global Change Biology 25, no. 12: 4147–4164. 10.1111/gcb.14811. 31449341

[gcb71123-bib-0086] Sauterey, B. , G. L. Gland , P. Cermeño , O. Aumont , M. Lévy , and S. M. Vallina . 2023. “Phytoplankton Adaptive Resilience to Climate Change Collapses in Case of Extreme Events—A Modeling Study.” Ecological Modelling 483: 110437. 10.1016/j.ecolmodel.2023.110437.

[gcb71123-bib-0087] Schaum, C. E. , and S. Collins . 2014. “Plasticity Predicts Evolution in a Marine Alga.” Proceedings of the Royal Society B: Biological Sciences 281, no. 1793: 20141486. 10.1098/rspb.2014.1486. PMC4173685 25209938

[gcb71123-bib-0088] Schlegel, R. , and A. Smit . 2018. “heatwaveR: A Central Algorithm for the Detection of Heatwaves and Cold‐Spells.” Journal of Open Source Software 3, no. 27: 821. 10.21105/joss.00821.

[gcb71123-bib-0089] Seebacher, F. , C. R. White , and C. E. Franklin . 2015. “Physiological Plasticity Increases Resilience of Ectothermic Animals to Climate Change.” Nature Climate Change 5, no. 1: 61–66. 10.1038/nclimate2457.

[gcb71123-bib-0090] Shiskin, J. 1967. The X‐11 Variant of the Census Method II Seasonal Adjustment Program. U.S. Department of Commerce, Bureau of the Census.

[gcb71123-bib-0091] Shultz, A. D. , Z. C. Zuckerman , and C. D. Suski . 2016. “Thermal Tolerance of Nearshore Fishes Across Seasons: Implications for Coastal Fish Communities in a Changing Climate.” Marine Biology 163, no. 4: 83. 10.1007/s00227-016-2858-2.

[gcb71123-bib-0092] Smale, D. A. , and T. Wernberg . 2013. “Extreme Climatic Event Drives Range Contraction of a Habitat‐Forming Species.” Proceedings of the Royal Society B: Biological Sciences 280, no. 1754: 20122829. 10.1098/rspb.2012.2829. PMC3574333 23325774

[gcb71123-bib-0093] Smale, D. A. , T. Wernberg , E. C. J. Oliver , et al. 2019. “Marine Heatwaves Threaten Global Biodiversity and the Provision of Ecosystem Services.” Nature Climate Change 9, no. 4: 306–312. 10.1038/s41558-019-0412-1.

[gcb71123-bib-0094] Smale, D. A. , A. L. E. Yunnie , T. Vance , and S. Widdicombe . 2015. “Disentangling the Impacts of Heat Wave Magnitude, Duration and Timing on the Structure and Diversity of Sessile Marine Assemblages.” PeerJ 3: e863. 10.7717/peerj.863. PMC4380158 25834773

[gcb71123-bib-0095] Smith, K. E. , M. T. Burrows , A. J. Hobday , et al. 2023. “Biological Impacts of Marine Heatwaves.” Annual Review of Marine Science 15, no. 1: 119–145. 10.1146/annurev-marine-032122-121437. 35977411

[gcb71123-bib-0096] Stock, C. , and J. Dunne . 2010. “Controls on the Ratio of Mesozooplankton Production to Primary Production in Marine Ecosystems.” Deep Sea Research Part I: Oceanographic Research Papers 57, no. 1: 95–112. 10.1016/j.dsr.2009.10.006.

[gcb71123-bib-0097] Stock, C. A. , J. P. Dunne , S. Fan , et al. 2020. “Ocean Biogeochemistry in GFDL's Earth System Model 4.1 and Its Response to Increasing Atmospheric CO_2_ .” Journal of Advances in Modeling Earth Systems 12, no. 10: e2019MS002043. 10.1029/2019MS002043.

[gcb71123-bib-0098] Stock, C. A. , J. P. Dunne , and J. G. John . 2014. “Global‐Scale Carbon and Energy Flows Through the Marine Planktonic Food Web: An Analysis With a Coupled Physical–Biological Model.” Progress in Oceanography 120: 1–28. 10.1016/j.pocean.2013.07.001.

[gcb71123-bib-0099] Stock, C. A. , J. G. John , R. R. Rykaczewski , et al. 2017. “Reconciling Fisheries Catch and Ocean Productivity.” Proceedings of the National Academy of Sciences 114, no. 8: E1441–E1449. 10.1073/pnas.1610238114. PMC5338393 28115722

[gcb71123-bib-0100] Tittensor, D. P. , H. K. Lotze , T. D. Eddy , et al. 2018. “ISIMIP2a Simulation Data From Fisheries & Marine Ecosystems (Fish‐MIP; Global) Sector (p. 1 Files) [Application/Octet‐Stream].” GFZ Data Services. 10.5880/PIK.2018.005.

[gcb71123-bib-0101] Tittensor, D. P. , C. Novaglio , C. S. Harrison , et al. 2021. “Next‐Generation Ensemble Projections Reveal Higher Climate Risks for Marine Ecosystems.” Nature Climate Change 11: 973–981. 10.1038/s41558-021-01173-9. PMC8556156 34745348

[gcb71123-bib-0102] Vantrepotte, V. , and F. Mélin . 2011. “Inter‐Annual Variations in the SeaWiFS Global Chlorophyll a Concentration (1997–2007).” Deep Sea Research Part I: Oceanographic Research Papers 58, no. 4: 429–441. 10.1016/j.dsr.2011.02.003.

[gcb71123-bib-0103] Verberk, W. C. E. P. , E. Henry , F. P. Leiva , V. Barbarossa , and A. M. Schipper . 2026. “Heat Tolerance and Its Plasticity in Freshwater and Marine Fishes Reflect Exposure to Extremes and Seasonal Variation in Habitat Temperatures.” Ecology Letters 29, no. 3: e70341. 10.1111/ele.70341. PMC12959617 41779621

