# Supplementary material for: Projecting Impacts of Marine Heatwaves and Their Effects on Long‐Term Changes in Marine Ecosystems Over the 21st Century
Source: Glob Chang Biol. 2026 Sep 27;32(10):e71123. doi: 10.1111/gcb.71123 (PMC13616748; doi:10.1111/gcb.71123)
Supplement: Supplementary file 1 [file GCB-32-e71123-s001.pdf]

Supplementary Appendix for

Projecting impacts of marine heatwaves and their effects on long-term changes in marine ecosystems over the 21st century

Appendix S1: EcoTroph-Dyn detailed

2.1 EcoTroph under steady-state condition

We used the EcoTroph modelling approach to study the effects of MHWs on marine ecosystems (du Pontavice et al., 2021; Gascuel, 2005; Gascuel et al., 2011; Gascuel & Pauly, 2009). This quasi-physical model represents the functioning of marine ecosystems as a continuous flow of biomass and energy surging up the food webs, from primary producers (low TLs) to top predators (high TLs). In EcoTroph, biomass enters the food web at trophic level TL = 1, corresponding to the photosynthetic activity of primary producers and recycling of nutrients by the microbial loop. Biomass at TLs higher than 2 comprises heterotrophic organisms, including herbivores, carnivores and omnivores such as fish, mammals, crabs, molluscs, and cephalopods. Fractional TLs result in a continuous distribution of biomass along TLs (considered here as consumers), defining the biomass trophic spectra (Gascuel et al., 2005). For computation simplification, in EcoTroph, the biomass spectrum is decomposed by an assembly of small TL classes, with each trophic class including all organisms within each class's lower and upper bond. The biomass spectrum in EcoTroph generally includes organisms across the water column living in habitats from pelagic to benthic.

EcoTroph computes the flow of biomass as continuous processes through the trophic spectrum, representing organisms' ontogenetic changes in TLs as they grow and abrupt jumps in TLs due to predation events. The aggregated biomass flows are described by a continuous function that represents the mean flow of biomass of individual organisms (Gascuel et al., 2008). The biomass flow in EcoTroph is represented by the traditional equations of fluid dynamics. Specifically, the continuous biomass flow, ϕ(τ), is described by:

$$\Phi(\tau) = B(\tau) \cdot \ K(\tau)\ , \qquad (1)$$

Where ϕ(τ) represents the biomass flow rate at TL τ, expressed in tons per year ($t \cdot year^{-1}$). It indicates the quantity of biomass moving through the TL due to processes such as predation or ontogeny. B(τ) denotes the density of biomass present at TL τ, measured in tons (t) per TL ($t \cdot TL^{-1}$). K(τ), the flow kinetic, is a rate coefficient, which could be interpreted as a turnover rate at TL τ, expressed

in TL per year (TL·year^{-1}). It describes the velocity at which the fraction of the biomass at that TL moves to the next level per unit of time. It thus measures the speed of the biomass flow in the food web, from low to high TLs, and is inversely proportional to biomass residence time. This latter corresponds to the time each organism stays at a given food web level, depending on its life expectancy.

A discrete approximation of the continuous distribution B(τ) over TLs is used for mathematical simplification. Hence, the model state variable becomes B_τ (the standing sock of biomass, in metric tons) present at a specific time under steady-state conditions within the TL class [τ, τ + Δτ[and Equation (1) becomes (see appendix S1 for details):

$$B_\tau = \frac{1}{K_\tau} \cdot \Phi_\tau \cdot \Delta\tau, \quad (2)$$

Where Φτ and Kτ are the mean biomass flow (in t·year^{-1}) and the mean flow kinetic (in TL·year^{-1}) within the trophic class [τ, τ + Δτ[, respectively.

Trophic transfers are usually faster at low TLs and in warm waters. Thus, the flow kinetic K_τ can be expressed as a function of TL, using the empirical equation of Gascuel et al. (2008), as:

$$K_\tau = 20.19 \cdot \tau_i^{-3.258} \cdot e^{0.041 \cdot SST_y} \;, \qquad (3)$$

Where SST_y corresponds to the annual sea surface temperature (SST) used as an input parameter (in this study equal to thermal class value, described in section 2.3.1), allowing us to calculate the flow kinetics for any theoretical biomes characterised by a known temperature

The biomass flow Φ(τ) is not conservative and can be expressed as decreasing function of TL such as (see details in Appendix S1):

$$\Phi(\tau + \Delta\tau) = \Phi(\tau) \cdot exp(-\mu_\tau) \cdot \Delta\tau) \;, \qquad (4)$$

where Φ(τ) is the biomass flow at TL=τ (i.e., at the start of the trophic class [τ, τ + Δτ[), and μ_τ (expressed in TL^{-1}) is the mean natural loss rate within the trophic class, representing the energy that does not get stored within a trophic class and is lost due to non-predation mortality, excretion, and respiration. Equation (4) also defines the transfer efficiency (TE) between continuous TLs, corresponding to the estimated fraction of biomass flow transferred from one TL to the next. TE can

be expressed as an empirical equation of the sea surface temperature (SST), according to du Pontavice et al. (2021):

$$TE = e^{-\mu_\tau} = e^{(-2.162+b+(-0.025+a)\cdot sst)} \cdot 1.038013\,, \quad (5)$$

where *a* and *b* are specific parameters for each biome type (du Pontavice et al., 2021) and SST the sea surface temperature of the time simulated.

Finally, EcoTroph defines the biomass flow Φ(τ) as the density of production at TL = τ. Therefore, the production P_τ of the trophic class [τ, τ + Δτ[is:

$$P_\tau = \int_{s=\tau}^{s=\tau+\Delta\tau'} \Phi(s) \cdot d\tau = \Phi_\tau \cdot \Delta\tau\ , \quad (6)$$

Hence, according to equations (2) and (6), EcoTroph highlights that biomass stems from the ratio of the production to the flow kinetic. Production is commonly expressed in t·year^{-1}, which implicitly refers to the conversion of biomass eaten at TL = τ − 1 into predator tissues whose mean TL is τ and thus does not explicitly refer to the TL in the production unit. Therefore, in a TL-based approach such as EcoTroph (wherein the width of trophic classes may differ from 1 TL), production must be expressed in t·TL·year^{-1}, thus ensuring full consistency of all units used.

In the steady-state version of the EcoTroph model, each trophic class have the same width, conventionally equal to 0.1 TL (Gasche et al., 2012; Gascuel, 2005) and a range starting at TL = 2 (corresponding to the first-order consumers), up to TL = 5.5, an appropriate range to cover all top predators in marine systems (Cortes, 1999; Pauly et al., 1998). This implies that the time needed for the biomass to flow from one to the next trophic class varies, from the low TLs usually characterised by fast trophic transfers to the high TLs marked by slow transfers.

2.2 Development of a dynamic version of EcoTroph

To examine the temporal dynamics of climatic events such as MHWs, we extended the steady-state EcoTroph to a time-dynamic model version (EcoTroph-Dyn). EcoTroph-Dyn simulated changes in biomass flows at a bi-weekly (15 days) time step to analyse how MHW-induced perturbation propagates over time through the modelled food web and to quantify its cumulative impacts. The

choice of a bi-weekly time step represents the average duration of most naturally occurring or experimentally simulated MHWs with observable ecological impacts (Smale et al., 2015).

In EcoTroph-Dyn, we made two main changes over the steady-state EcoTroph to enable the model to undertake time-dynamic simulations of the trophic effects of MHWs. These include changes in modelling flow kinetics and boundaries of trophic classes, as well as the representation of biomass flow in the basic equations.

2.2.1 Flow kinetics and boundaries of trophic classes

Biomass spectra in EcoTroph-Dyn were composed of trophic classes with variable widths of TLs. The width of trophic classes [τ_i; τ_{i+1}[in modelled biomass spectra were determined based on the estimated mean flow kinetics so biomass could transfer up a trophic spectrum in each time step (Δt = 1/26 year). Mean flow kinetics is defined as the speed of the trophic flow through the food web and is expressed in $TL.y^{-1}$. Thus:

$$\tau_{i+1} = \tau_i + K\tau \cdot \Delta t \ , \qquad (7)$$

where $\tau_i \in [2; 5.5]$, $\tau_{i=0}$=2, and Kτ defined by empirical equation (3)

This equation allows us to define the trophic class boundaries in a reference state, characterised by a baseline mean water temperature for each modelled biomass spectrum . The baseline mean water temperature is defined as the average sea surface temperature from 1st January 1982 to 31st December 2011 (see section 2.3.1). When water temperature changes from the baseline, the estimated flow kinetics of the biomass spectrum and the trophic class boundaries are re-computed.

Moreover, MHWs lead to the death of marine organisms, affecting their life expectancy(Smith et al., 2023). In EcoTroph-Dyn, changes in the life expectancy of organisms are represented by the changes in loss rate in the biomass spectrum, i.e. the proportion of the biomass that does not stay or move up in the food web (du Pontavice et al., 2021; Gascuel et al., 2008). To account for the MHW's effects on loss rate, the flow kinetic and the resulting width of the trophic class were computed as follows:

$$\tau_{i+1} - \tau_i = K\tau \cdot \Delta t = 20.19 \cdot \tau_i^{-3.258} \cdot e^{0.041 \cdot SST_y} \cdot (1 + \eta_\tau) \cdot \Delta t, \quad (8)$$

where η_τ is the MHW-associated additional loss rate, defined according to the method described in section 2.3.2.

Equation (8) implies that any warming and any MHW's additional losses in the trophic flow reduce the life expectancy of organisms and thus accelerate trophic transfers towards higher TLs or detritus. At each time step, the upper and lower TLs of every trophic class were recalculated given the environmental temperature (SST) that the biomass spectrum is exposed to at time step y and the MHW-associated losses (η_τ).

1. <u>2.2.2 Modelling biomass flow in dynamic simulations</u>

EcoTroph-Dyn represents time-dynamic changes in biomass flow by (step 1) computing biomass flow between trophic classes and (step 2) calculating biomass at each trophic class for each time step . To calculate biomass flow (Φ) from trophic class τ to $\tau + \Delta\tau'$ dynamically, we modified equation 4.

$$\Phi(\tau + \Delta\tau', t + 1) = \Phi(\tau, t) \cdot exp(-(\mu_t + \eta_{\tau,t}) \cdot \Delta\tau_{,t}) \quad (9)$$

where μ_τ (expressed in TL^{-1}, equation (5)) represents the mean natural losses within the trophic class through non-predation mortality, excretion, and respiration over a trophic class interval. The $\eta_{\tau,t}$ parameter (expressed in TL^{-1}) represents the mean loss rate caused by the instantaneous mortality of species in each trophic class induced by the MHW event.

In step 2, as the trophic class intervals (τ and τ+Δτ) of each biomass spectra are re-computed at every time step, the production is redistributed into the new trophic class between each time step. The production is allocated proportionally according to the width of the trophic classes. This can be expressed as:

$$P_{\tau'} = \sum_{\tau'_i}^{\tau'_{i+1}} Fraction \cdot P_\tau \quad (10)$$

With case 1: $\tau'_{i+1} \geq \tau_{i+1}$:

$$Fraction = \frac{\tau_{i+1} - max(\tau_i; \tau'_i)}{\tau_{i+1} - \tau_i}$$

case 2: $\tau'_{i+1} \leq \tau_{i+1}$:

$$Fraction = \frac{\tau'_{i+1} - max(\tau_i;\ \tau'_i)}{\tau_{i+1} - \tau_i}$$

Finally, in each trophic class [τ, τ+Δτ'[the mean biomass can be expressed as:

$$B_{\tau,t} = \frac{P_{\tau,t}}{K_{\tau,t}} \quad \text{or} \quad \frac{P_{\tau',t}}{K_{\tau,t}} \quad \text{if boundaries change} \qquad \text{Equation (2, dyn)}$$

Appendix S2: Transfer efficiency of higher trophic levels

Table S2: Specific parameters to estimate trophic transfer efficiency for each type of ecosystem (equation (9), in the Methods section).

Ecosystem type	Trophic transfer efficiency coefficient	
	**a**	**b**
**Polar**	0	0
**Temperate**	-0.013	0.142
**Tropical**	0.015	-0.352
**Upwelling**	-0.032	0.167

Appendix S3: ESM and NOAA AVHRR SST' time series

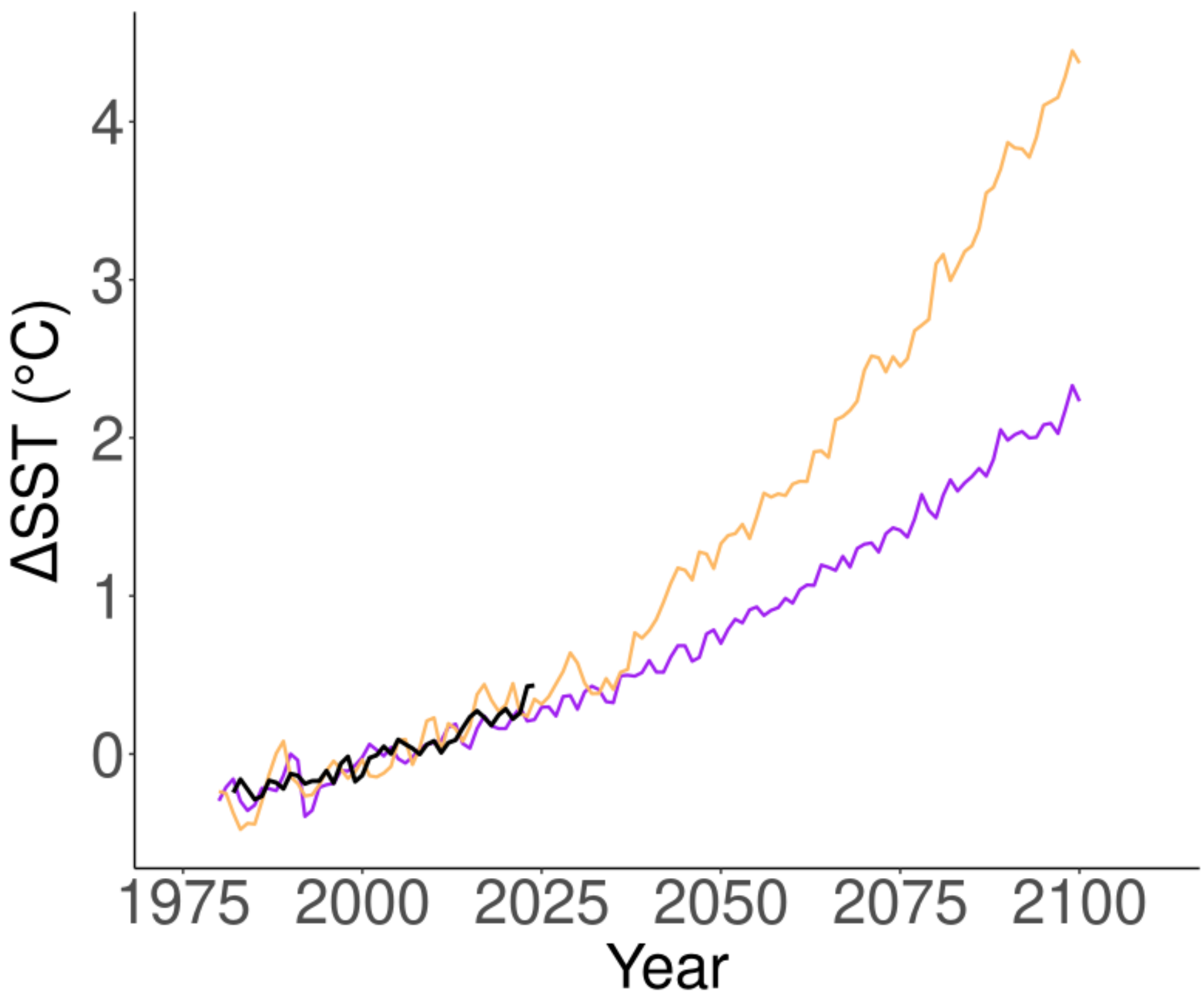

Figure S3: Oserved and projected change in sea surface temperature. NOAA AVHRR observation-based estimates (black lines) and projected SSP5-8.5 scenario (IPSL=orange, GFDL=purple) global changes over the period of 1980 to 2100 relative to the 1994–2014 period.

Appendix S4: MHW loss rate algorithm computation

- MHW characterisation and detection from 1982 to 2021

To characterise MHW in each ocean spatial cell, we analysed daily SST observations from the NOAA's AVHRR data (Reynolds et al., 2007; https://www.ncei.noaa.gov/access/metadata/landing-page/bin/iso?id=gov.noaa.ncdc:C00680). We defined MHW as a discrete prolonged anomalously warm water event when the daily SSTs exceed an extreme temperature threshold value for at least five consecutive days (Hobday et al., 2016). The extreme temperature threshold value was calculated for each 1° latitude x 1° longitude spatial cell as the 90th percentile of daily SST from the 30-year historical time series from January 1982 to December 2011. We did not calculate threshold values by season; thus, MHW events were identified by a single threshold across the year. As a result, we detected MHWs mostly occurring during the year's warmest months (see Figure S4a for schematic explanation). This approach to identifying the MHW threshold represents biological extreme temperature in the local (spatial cell) context. It is appropriate to assess the direct mortality associated with MHWs (Oliver et al., 2021). We determined a reference average sea surface temperature (SST average) for each spatial cell by analysing data from the 1st of January 1982 to the 31st of December 2011. Utilising this reference average SST, we classified each spatial cell into 'thermal classes,' with each class representing a 1°C increment of the reference SST average ranging from -1°C to 29°C (refer to Figure S1b). We used the R package heatwaveR described at https://robwschlegel.github.io/heatwaveR/ to compute MHWs characteristics in each spatial cell from January 1982 to December 2021. Thus, we finally obtained MHW characteristics for each 1° per 1° of longitude and latitude ocean cell up to December 2021. The considered MHW characteristics are the threshold value defining MHWs, MHW's duration (in days), category and intensity (mean SST anomaly) and declaration of MHW days over the SST time series.

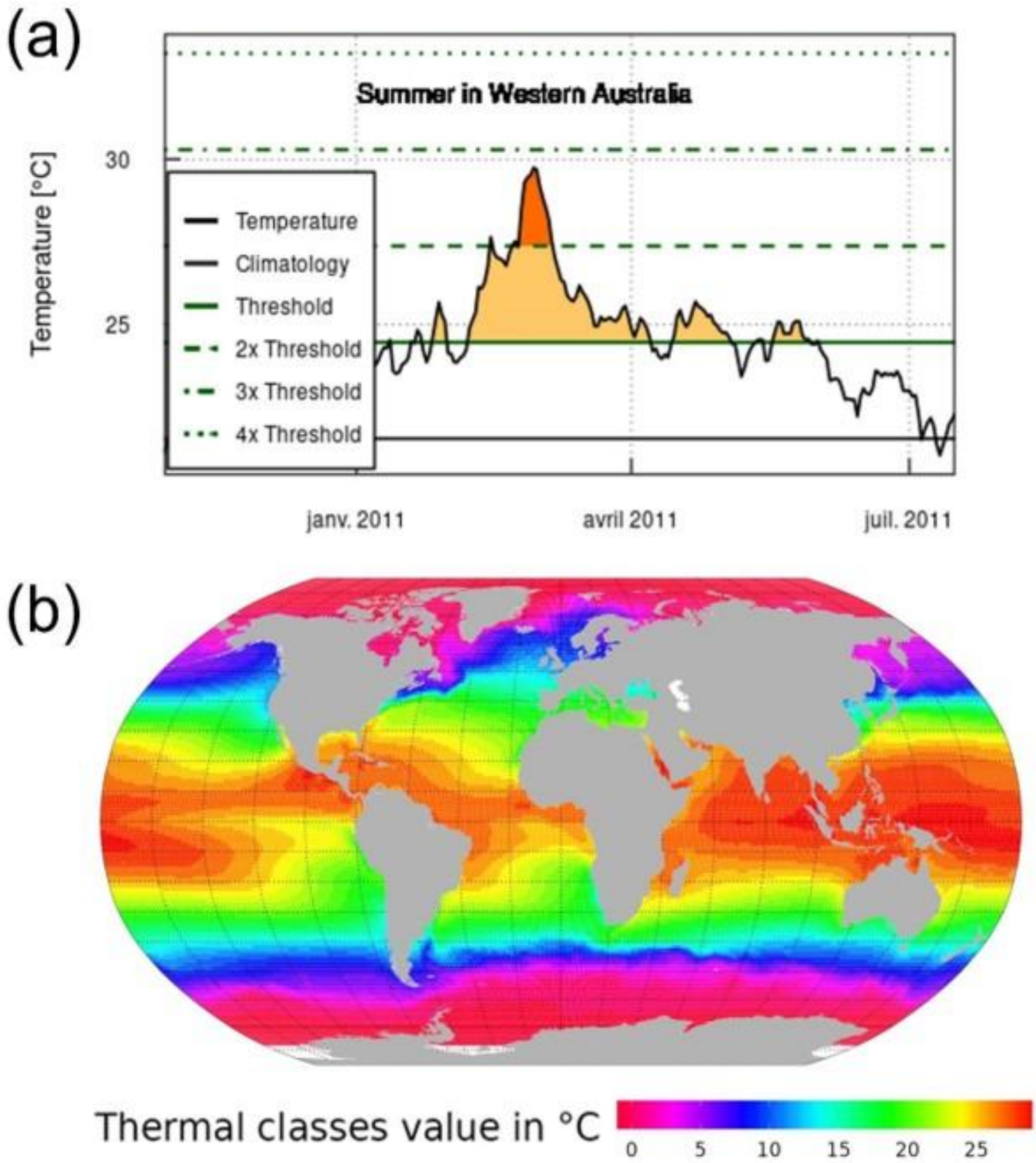

Figure S4a: Marine heatwave detection method and reference average SST of each ocean cell. (a) Schematic explanation of MHWs detection for a spatial cell. The solid horizontal green and black lines represented the extreme threshold value and the reference temperature, respectively. (b) A map of thermal classes of 1o C intervals from -1 to 29°C categorised based on the reference temperature at each spatial cell over the period from 1st January 1982 to 31st December 2011. Map lines delineate study areas and do not necessarily depict accepted national boundaries

- Estimation of species distribution and associated thermal niche

We developed an algorithm depending on the MHWs' characteristics to express the loss rate of trophic transfers associated with MHW. First, we identified a list of marine species with their occurrence records (3242 bivalves, 500 cephalopods species, 3116 crabs species, and 12782 fish species) gathered from the publicly accessible databases: OBIS (www.iobis.org), the Intergovernmental Oceanographic Commission (ioc-unesco.org), GBIF (www.gbif.org), Fishbase (www.fishbase.org), and the International Union for the Conservation of Nature (http://www.iucnredlist.org/technical-documents/spatial-data). We then cleaned the data by removing duplicate entries, terrestrial occurrences, and occurrences outside the known species habitat from the aggregated species occurrence dataset (Froese & Pauly, 2018). Additionally, we excluded zooplankton from the algorithm development due to limited evidence of direct mortality induced by Marine Heatwaves (MHWs), with observed responses mainly manifesting as range shifts and alterations in community structure (Arimitsu et al., 2021; Suryan et al., 2021; Winans et al., 2023). Marine mammals and seabirds were also omitted from algorithm development, as their mortality linked to MHWs primarily stems from secondary effects such as diminished quality and quantity of food supply (Cavole et al., 2016; Piatt et al., 2020) rather than direct heat stress impact. Subsequently, the data were rasterised into a grid covering the global oceans (1° longitude by 1° latitude), denoting the historical presence of each species. Species with occurrence records in fewer than 30 cells were excluded from further analysis (Hernandez et al., 2006).

In a second step, we utilised an ensemble species distribution modelling approach (Asch et al., 2018; Reygondeau, 2019) at a 1° grid scale. Four environmental niche models (ENMs) were applied: Bioclim, Boosted Regression Trees models (Thuiller et al., 2009), Maxent (Phillips et al., 2006), and the Non-Parametric Probabilistic Ecological Niche model (Beaugrand et al., 2011), using global climatology satellite data (AVHRR). Model accuracy was assessed using the area under the curve (AUC) analysis of the receiver operating characteristic (ROC), discarding models with AUC below 0.5 (Sing et al., 2005). The evaluation employed the pROC package in R (Robin et al., 2011). We then calculated the average

Habitat Suitability Index (HSI) weighted by the AUC values of each ENM for each spatial cell and species. An HSI threshold for each species was estimated using their prevalence. Spatial cells with HSI below the threshold were deemed non-viable habitats.

Species' predicted thermal niches were quantified from spatial distributions using averaged satellite sea surface temperature (SST) data (AVHRR) from 1982 to 2011. The average SST from 1982 to 2011 was recorded for each spatial cell above the HSI threshold. We then characterised the predicted thermal niche (histogram of all the average values of SST), and more specifically, the upper-temperature threshold, for each species from the 95th percentile of the SST records where they were predicted to occur.

- Additional loss rate associated with MHW

For each spatial cell belonging to each thermal class, we calculated the percentage of species in each trophic class exposed to thermal stress induced by MHWs intensity above their estimated temperature threshold (95th percentile of their thermal niche). Matching MHW intensity in each spatial cell from 1981 to 2021 with species' temperature thresholds, we determined the percentage of species exposed to temperatures exceeding their thresholds. Thermal stress of a species was assumed dependent on MHW category (1 to 4, based on SST anomaly) and species' trophic level (<2.5, 2.5-3.0, 3.0-3.5, 3.5-4.0, 4.0-4.5, 4.5-5.0, >5.0), estimated from FishBase and SeaLifeBase.

To obtain a continuous representation of the percentage of species undergoing a thermal stress as the intensity of MHW increases, we decided to transform the discrete MHW categorisation (Hobday et al., 2018) to a continuous MHW intensity index as follows:

$$MHW_{cat,i} = \frac{MHW\ mean\ anomaly,i}{cat1\ associated\ anomaly,i} \quad , (11)$$

MHW mean anomaly was calculated as the difference between the MHW mean SST anomaly and the reference temperature of each spatial cell, and “cat1 associated anomaly” is the mean threshold value used to identify category 1 MHWs in each spatial cell.

We fit the estimated percentage of species undergoing thermal stress with the MHW intensity index and species' trophic class to a nonlinear function. A Gompertz function was selected after preliminary tests because it is better fitted to data than logistic or other mathematical functions with similar shapes. The Gompertz function is expressed as:

$$Percentage\ of\ species\ undergoing\ a\ thermal\ stress = exp^{-exp^{b\_tl_i \cdot (MHW_{cat} - lt50\_tl_i)}},\quad (12);$$

We estimated the parameters b_tl_i, $lt50\_tl_i$, and $MHW_{cat,i}$ for each thermal class i. The parameters b_tl_i and $lt50\_tl_i$ correspond to the slope of the function and the index of marine heatwave intensity (MHW_{cat}) at which 50% of the species are undergoing thermal stress, respectively.

For each thermal class i, parameters b_tl_i and $lt50\_tl_i$ were expressed as (figure S4b) :

$b\_tl_i = -1.4511 - e^{0.4223 \cdot (i - 22.4926)}$, (12) and

$lt50\_tl_i = 3.29 - 0.485 \cdot i + 0.0306 \cdot i^2 - 0.000608 \cdot i^3$ when trophic level <2.5 and

$lt50\_tl_i = 3.55 - 0.271 \cdot i + 0.014 \cdot i^2 - 0.000304 \cdot i^3$ when trophic level >=2.5. (13)

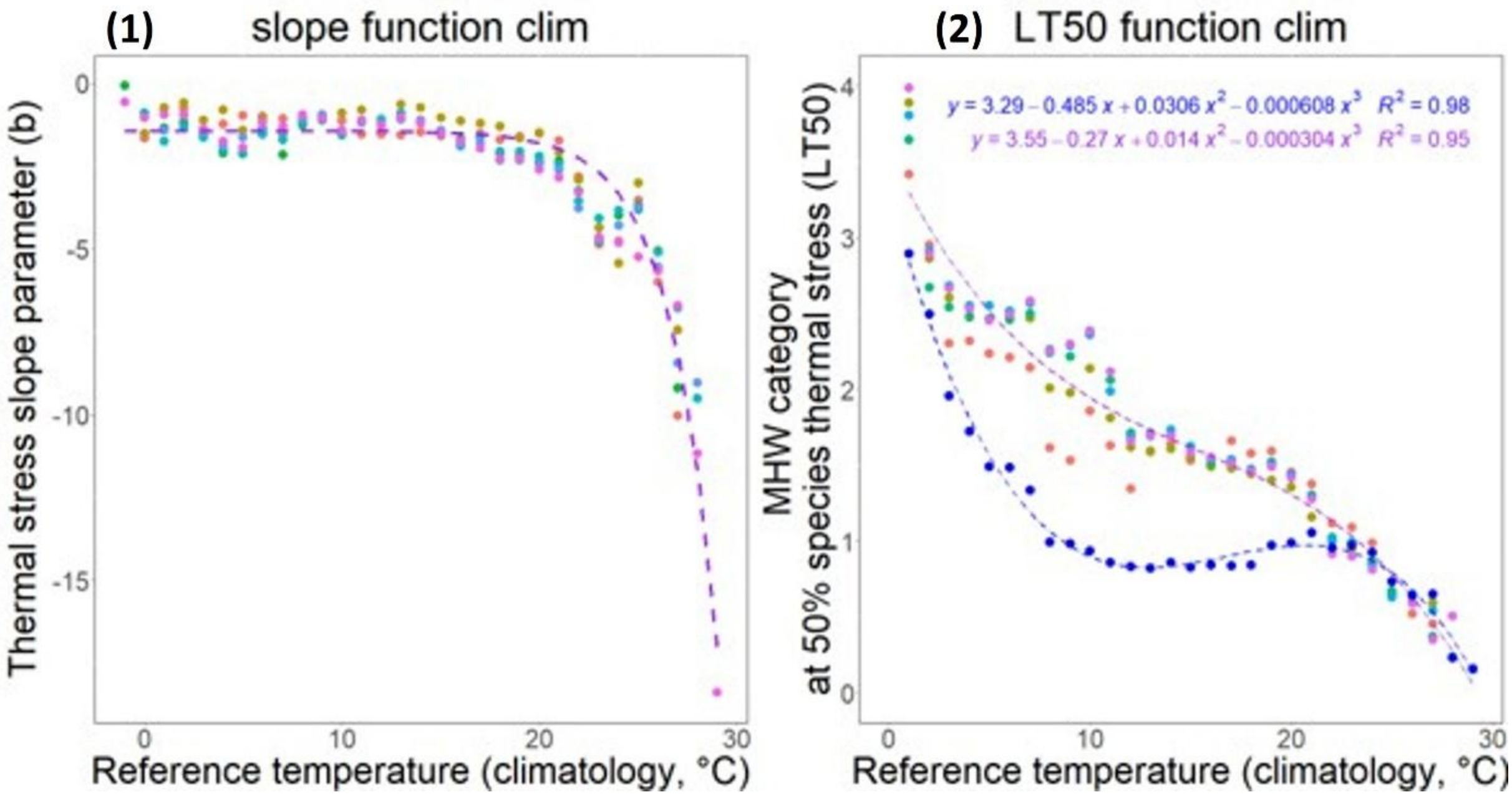

Figure S4b: Parameters of each reference temperature individual thermal stress algorithm model. With (1) The Gompertz slope parameter (b) at the inflection point, representing the rate of thermal stress acceleration. (2) The marine heatwave (MHW) category causing 50% of species to undergo

thermal stress (LT_{50}). Blue curves/points correspond to lower trophic levels (TL < 2.5), while other colors represent higher trophic levels (TL ≥ 2.5). Each point represents a specific trophic level coefficient value, with six distinct trophic levels plotted per reference temperature (climatology) baseline value.

Finally, to move from the thermally stressed stage to the loss rate (η_i), we assumed that species were continuously challenged by MHW increased intensity (Figure S4c) for schematic differences) expressed as.

$$\eta_i = exp^{-exp^{b_i \cdot \alpha \cdot \left(MHW_{cat,i} - \frac{lt50_i}{\alpha}\right)}} \cdot \beta, (14)$$

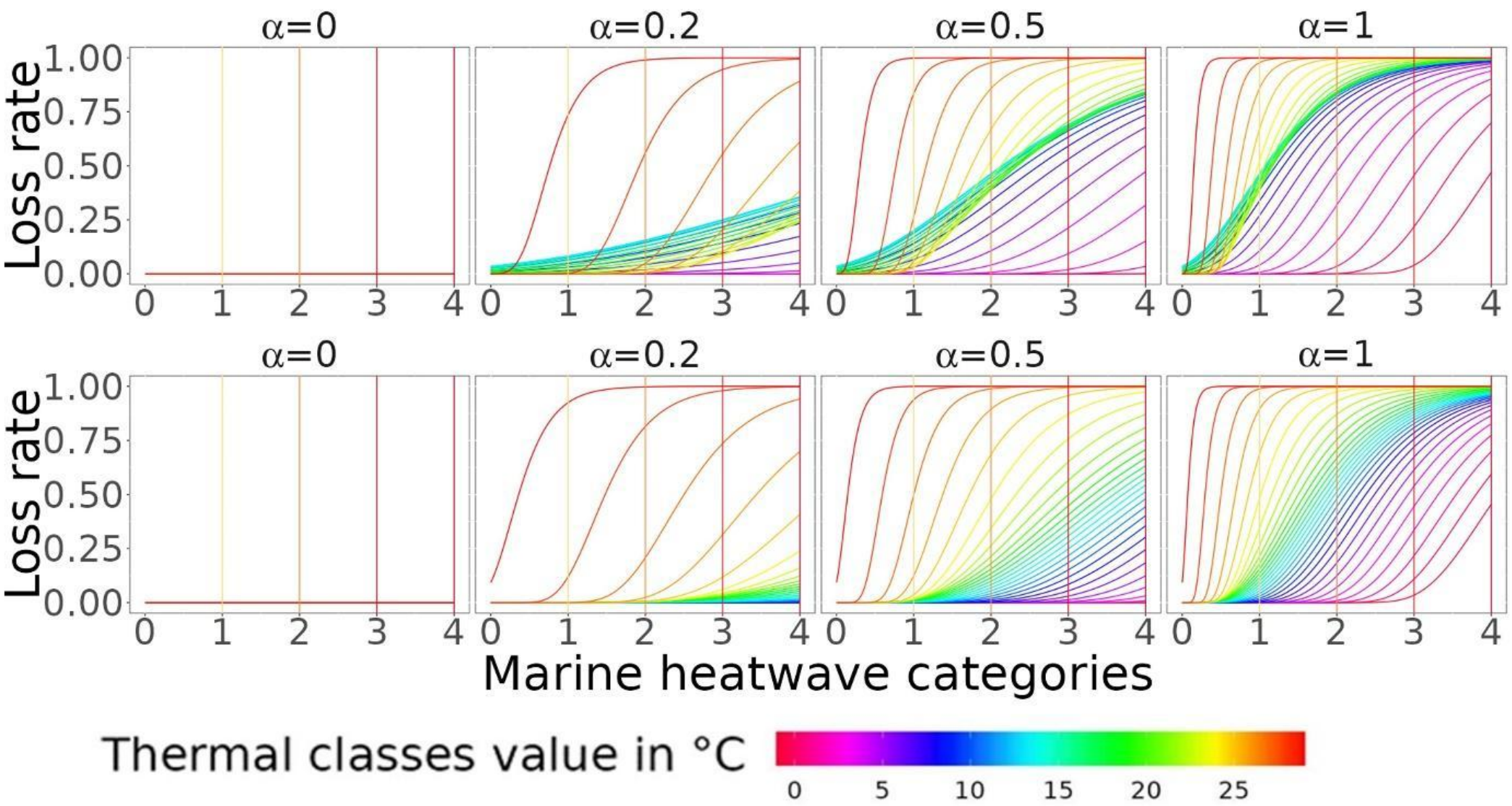

Figure S4c: loss rate associated with MHWs occurrences. With the ecological hypothesis that MHW increases in intensity/category continuously challenge the aggregated response across species. On the plots, b is equal to 1. The top and bottom rows correspond to the loss rate of trophic level <2.5 and trophic level>=2.5, respectively.

We explored the sensitivity of the results to species' acclimation capacity to MHW conditions by assuming that acclimation reduces the mortality rate due to species' exposure to thermal stress. We tested four acclimation capacity settings represented by the values of the coefficient α. These settings are full acclimation (α = 0; no mortality due to thermal stress and set $\eta_i = 0$), partial acclimation (α = 0.2, 0.5; 20%, and 50% of the species die because of thermal stress, respectively) and no

acclimation (α =1; all species die when they are under thermal stress). We also related the loss rate to the MHW duration over the fortnight by assuming that the duration increases the mortality rate. The duration of MHW is represented by β and ranges from β=0; no MHW to β=1; MHW lasting 15 days of the fortnight (see section 2.3.2 for β computation).

Bibliography of appendix 1 & 4

Arimitsu, M. L., Piatt, J. F., Hatch, S., Suryan, R. M., Batten, S., Bishop, M. A., Campbell, R. W., Coletti, H., Cushing, D., Gorman, K., Hopcroft, R. R., Kuletz, K. J., Marsteller, C., McKinstry, C., McGowan, D., Moran, J., Pegau, S., Schaefer, A., Schoen, S., … Biela, V. R. (2021). Heatwave-induced synchrony within forage fish portfolio disrupts energy flow to top pelagic predators. *Global Change Biology*, *27*(9), 1859–1878. https://doi.org/10.1111/gcb.15556

Asch, R. G., Cheung, W. W. L., & Reygondeau, G. (2018). Future marine ecosystem drivers, biodiversity, and fisheries maximum catch potential in Pacific Island countries and territories under climate change. *Marine Policy*, *88*, 285–294. https://doi.org/10.1016/j.marpol.2017.08.015

Beaugrand, G., Lenoir, S., Ibañez, F., & Manté, C. (2011). A new model to assess the probability of occurrence of a species, based on presence-only data. *Marine Ecology Progress Series*, *424*, 175–190. https://doi.org/10.3354/meps08939

Cavole, L., Demko, A., Diner, R., Giddings, A., Koester, I., Pagniello, C., Paulsen, M.-L., Ramirez-Valdez, A., Schwenck, S., Yen, N., Zill, M., & Franks, P. (2016). Biological Impacts of the 2013–2015 Warm-Water Anomaly in the Northeast Pacific: Winners, Losers, and the Future. *Oceanography*, *29*(2). https://doi.org/10.5670/oceanog.2016.32

Cortes, E. (1999). Standardized diet compositions and trophic levels of sharks. *ICES Journal of Marine Science*, *56*(5), 707–717. https://doi.org/10.1006/jmsc.1999.0489

du Pontavice, H., Gascuel, D., Reygondeau, G., Stock, C., & Cheung, W. W. L. (2021). Climate-induced decrease in biomass flow in marine food webs may severely affect predators and ecosystem production. *Global Change Biology*, *27*(11), 2608–2622. https://doi.org/10.1111/gcb.15576

Froese, R., & Pauly, D. (2018). *World Wide Web Electron. Publ*. URL www.fishbase.org

Gasche, L., Gascuel, D., Shannon, L., & Shin, Y.-J. (2012). Global assessment of the fishing impacts on the Southern Benguela ecosystem using an EcoTroph modelling approach. *Journal of Marine Systems*, *90*(1), 1–12. https://doi.org/10.1016/j.jmarsys.2011.07.012

Gascuel, D. (2005). The trophic-level based model: A theoretical approach of fishing effects on marine ecosystems. *Ecological Modelling*, *189*(3–4), 315–332. https://doi.org/10.1016/j.ecolmodel.2005.03.019

Gascuel, D., Bozec, Y.-M., Chassot, E., Colomb, A., & Laurans, M. (2005). The trophic spectrum: Theory and application as an ecosystem indicator. *ICES Journal of Marine Science*, *62*(3), 443–452. https://doi.org/10.1016/j.icesjms.2004.12.013

Gascuel, D., Guénette, S., & Pauly, D. (2011). The trophic-level-based ecosystem modelling approach: Theoretical overview and practical uses. *ICES Journal of Marine Science*, *68*(7), 1403–1416. https://doi.org/10.1093/icesjms/fsr062

Gascuel, D., Morissette, L., Palomares, M. L. D., & Christensen, V. (2008). Trophic flow kinetics in marine ecosystems: Toward a theoretical approach to ecosystem functioning. *Ecological Modelling*, *217*(1–2), 33–47. https://doi.org/10.1016/j.ecolmodel.2008.05.012

Gascuel, D., & Pauly, D. (2009). EcoTroph: Modelling marine ecosystem functioning and impact of fishing. *Ecological Modelling*, *220*(21), 2885–2898. https://doi.org/10.1016/j.ecolmodel.2009.07.031

Hernandez, P. A., Graham, C. H., Master, L. L., & Albert, D. L. (2006). The effect of sample size and species characteristics on performance of different species distribution modeling methods. *Ecography*, *29*(5), 773–785. https://doi.org/10.1111/j.0906-7590.2006.04700.x

Hobday, A., Alexander, L. V., Perkins, S. E., Smale, D. A., Straub, S. C., Oliver, E. C. J., Benthuysen, J. A., Burrows, M. T., Donat, M. G., Feng, M., Holbrook, N. J., Moore, P. J., Scannell, H. A., Sen Gupta, A., & Wernberg, T. (2016). A hierarchical approach to defining marine heatwaves. *Progress in Oceanography*, *141*, 227–238. https://doi.org/10.1016/j.pocean.2015.12.014

Oliver, E. C. J., Benthuysen, J. A., Darmaraki, S., Donat, M. G., Hobday, A. J., Holbrook, N. J., Schlegel, R. W., & Sen Gupta, A. (2021). Marine Heatwaves. *Annual Review of Marine Science*, *13*(1), 313–342. https://doi.org/10.1146/annurev-marine-032720-095144

Pauly, D., Christensen, V., Dalsgaard, J., Froese, R., & Torres, F. (1998). Fishing Down Marine Food Webs. *Science*, *279*(5352), 860–863. https://doi.org/10.1126/science.279.5352.860

Phillips, S. J., Anderson, R. P., & Schapire, R. E. (2006). Maximum entropy modeling of species geographic distributions. *Ecological Modelling*, *190*(3–4), 231–259. https://doi.org/10.1016/j.ecolmodel.2005.03.026

Piatt, J. F., Parrish, J. K., Renner, H. M., Schoen, S. K., Jones, T. T., Arimitsu, M. L., Kuletz, K. J., Bodenstein, B., García-Reyes, M., Duerr, R. S., Corcoran, R. M., Kaler, R. S. A., McChesney, G. J., Golightly, R. T., Coletti, H. A., Suryan, R. M., Burgess, H. K., Lindsey, J., Lindquist, K., … Sydeman, W. J. (2020). Extreme mortality and reproductive failure of common murres resulting from the northeast Pacific marine heatwave of 2014-2016. *PLOS ONE*, *15*(1), e0226087. https://doi.org/10.1371/journal.pone.0226087

Reygondeau, G. (2019). Current and future biogeography of exploited marine groups under climate change. In *Predicting Future Oceans* (pp. 87–101). Elsevier. https://doi.org/10.1016/B978-0-12-817945-1.00009-5

Robin, X., Turck, N., Hainard, A., Tiberti, N., Lisacek, F., Sanchez, J.-C., & Müller, M. (2011). pROC: An open-source package for R and S+ to analyze and compare ROC curves. *BMC Bioinformatics*, *12*(1), 77. https://doi.org/10.1186/1471-2105-12-77

Sing, T., Sander, O., Beerenwinkel, N., & Lengauer, T. (2005). ROCR: Visualizing classifier performance in R. *Bioinformatics*, *21*(20), 3940–3941. https://doi.org/10.1093/bioinformatics/bti623

Smale, D. A., Yunnie, A. L. E., Vance, T., & Widdicombe, S. (2015). *Disentangling the impacts of heat wave magnitude, duration and timing on the structure and diversity of sessile marine assemblages*.

Smith, K. E., Burrows, M. T., Hobday, A. J., King, N. G., Moore, P. J., Sen Gupta, A., Thomsen, M. S., Wernberg, T., & Smale, D. A. (2023). Biological Impacts of Marine Heatwaves. *Annual Review of Marine Science*, *15*(1), 119–145. https://doi.org/10.1146/annurev-marine-032122-121437

Suryan, R. M., Arimitsu, M. L., Coletti, H. A., Hopcroft, R. R., Lindeberg, M. R., Barbeaux, S. J., Batten, S. D., Burt, W. J., Bishop, M. A., Bodkin, J. L., Brenner, R., Campbell, R. W., Cushing, D. A., Danielson, S. L., Dorn, M. W., Drummond, B., Esler, D., Gelatt, T., Hanselman, D. H., … Zador, S. G. (2021). Ecosystem response persists after a prolonged marine heatwave. *Scientific Reports*, *11*(1), 6235. https://doi.org/10.1038/s41598-021-83818-5

Thuiller, W., Lafourcade, B., Engler, R., & Araújo, M. B. (2009). BIOMOD – a platform for ensemble forecasting of species distributions. *Ecography*, *32*(3), 369–373. https://doi.org/10.1111/j.1600-0587.2008.05742.x

Winans, A. K., Herrmann, B., & Keister, J. E. (2023). Spatio-temporal variation in zooplankton community composition in the southern Salish Sea: Changes during the 2015–2016 Pacific marine heatwave. *Progress in Oceanography*, *214*, 103022. https://doi.org/10.1016/j.pocean.2023.103022

Appendix S5: Study area

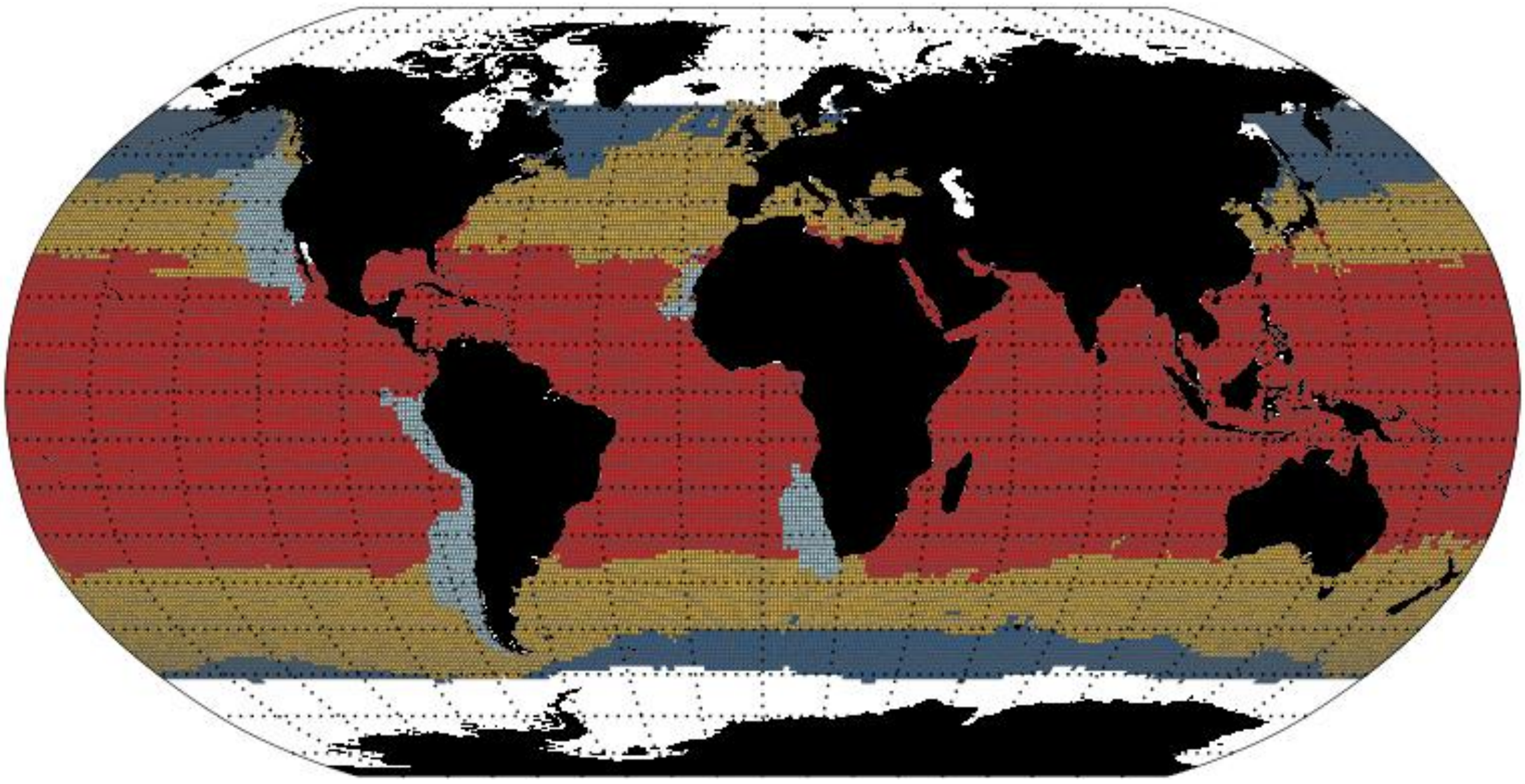

Figure S5: Map of where EcoTroph-Dyn have been applied and the associated biome types. The colours refer to the biome types; with temperate, tropical, upwelling, and polar corresponding to orange, red, light blue, and blue, respectively. Map lines delineate study areas and do not necessarily depict accepted national boundaries

Appendix S6: time series decomposition

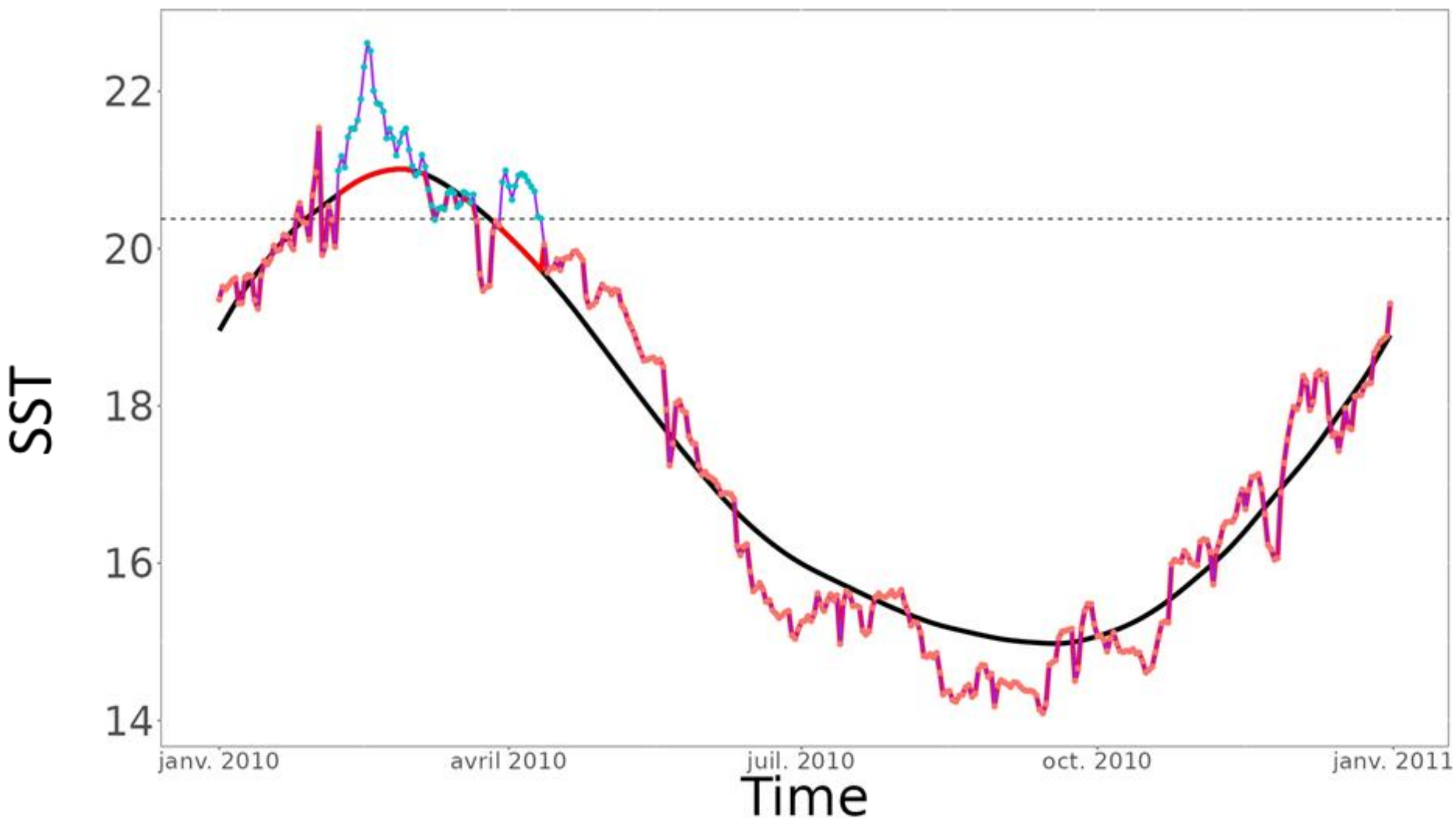

Figure S6: Illustration of removing MHWs from the time series on each ocean cell. Blue and red points correspond to MHW days and non-MHW days, respectively. The SST original time series Y_t, the SST time series once MHWs are removed, and the long-term linear trend plus the seasonal component ($T_t + S_t$) correspond to the purple thin line, red thick line, and black thick line, respectively. Finally, the dotted grey line corresponds to the fixed threshold value for MHWs detection in the specific ocean cell taken for the illustration.

Appendix S7: Number of MHWs days associated with a 2°C warming level above preindustrial levels (1850-1900)

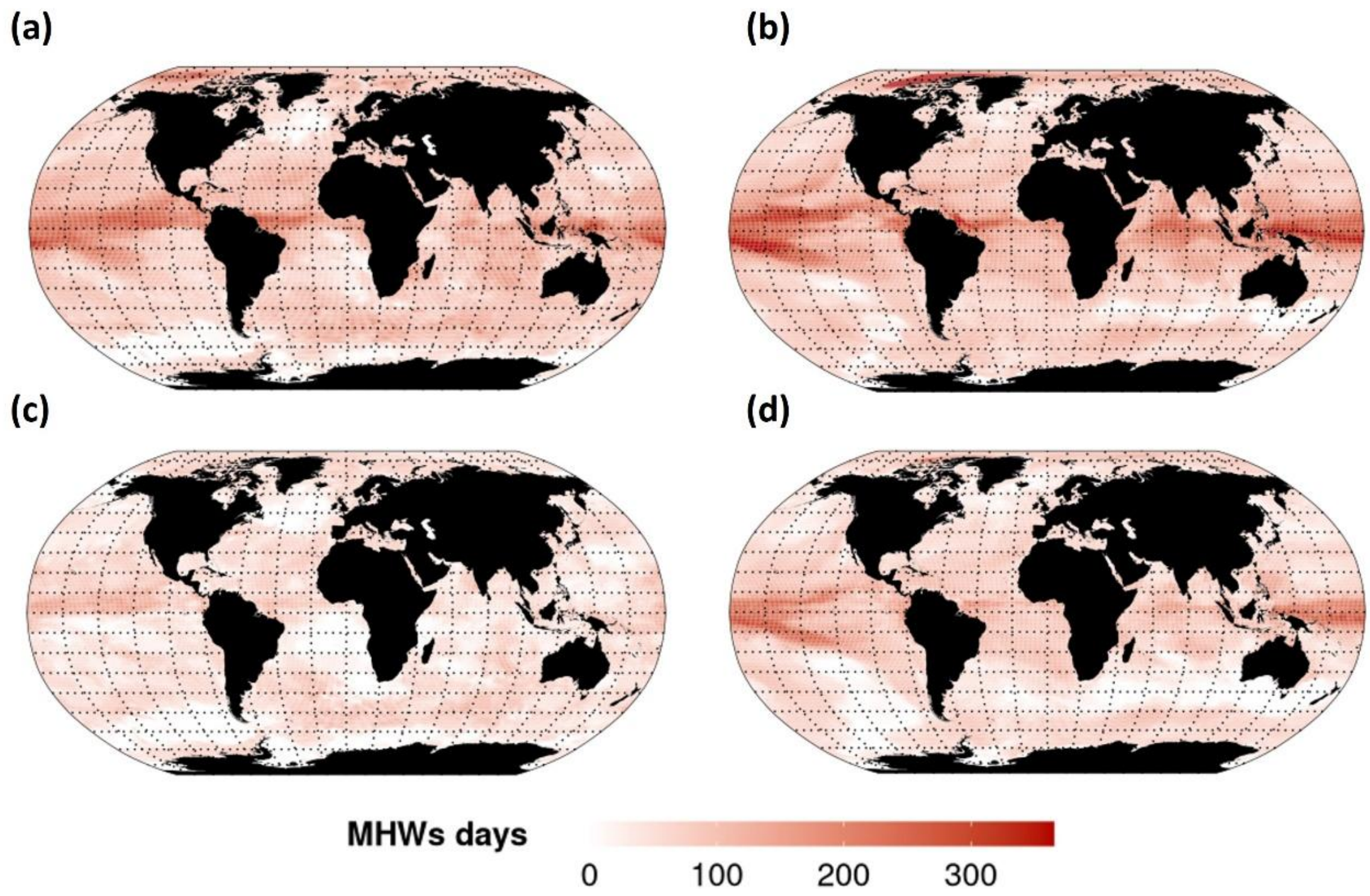

Figure S7: Number of MHWs days associated with a +2°C global warming level above preindustrial level (1850-1900). Maps (a) and (c) correspond to the number of MHWs days according to GFDL fixed and moving baseline, respectively. Maps (b) and (d) correspond to the number of MHWs days according to IPSL fixed and moving baseline, respectively. Map lines delineate study areas and do not necessarily depict accepted national boundaries

Table S7: Number of MHW days per biome associated with a +2°C global warming level above the pre-industrial baseline (1850–1900).

ESM	Baseline	Biome	Average number of MHW days
IPSL	Fix	Polar	90
IPSL	Fix	Temperate	79
IPSL	Fix	Tropical	128
IPSL	Fix	Upwelling	99
IPSL	Moving	Polar	71
IPSL	Moving	Temperate	48
IPSL	Moving	Tropical	89
IPSL	Moving	Upwelling	76
GFDL	Fix	Polar	88
GFDL	Fix	Temperate	89
GFDL	Fix	Tropical	112
GFDL	Fix	Upwelling	76
GFDL	Moving	Polar	55
GFDL	Moving	Temperate	55
GFDL	Moving	Tropical	52
GFDL	Moving	Upwelling	47

Appendix S8: biomes' biomass flow parameters responses to MHWs occurrence under global warming level above preindustrial level (1850-1900)

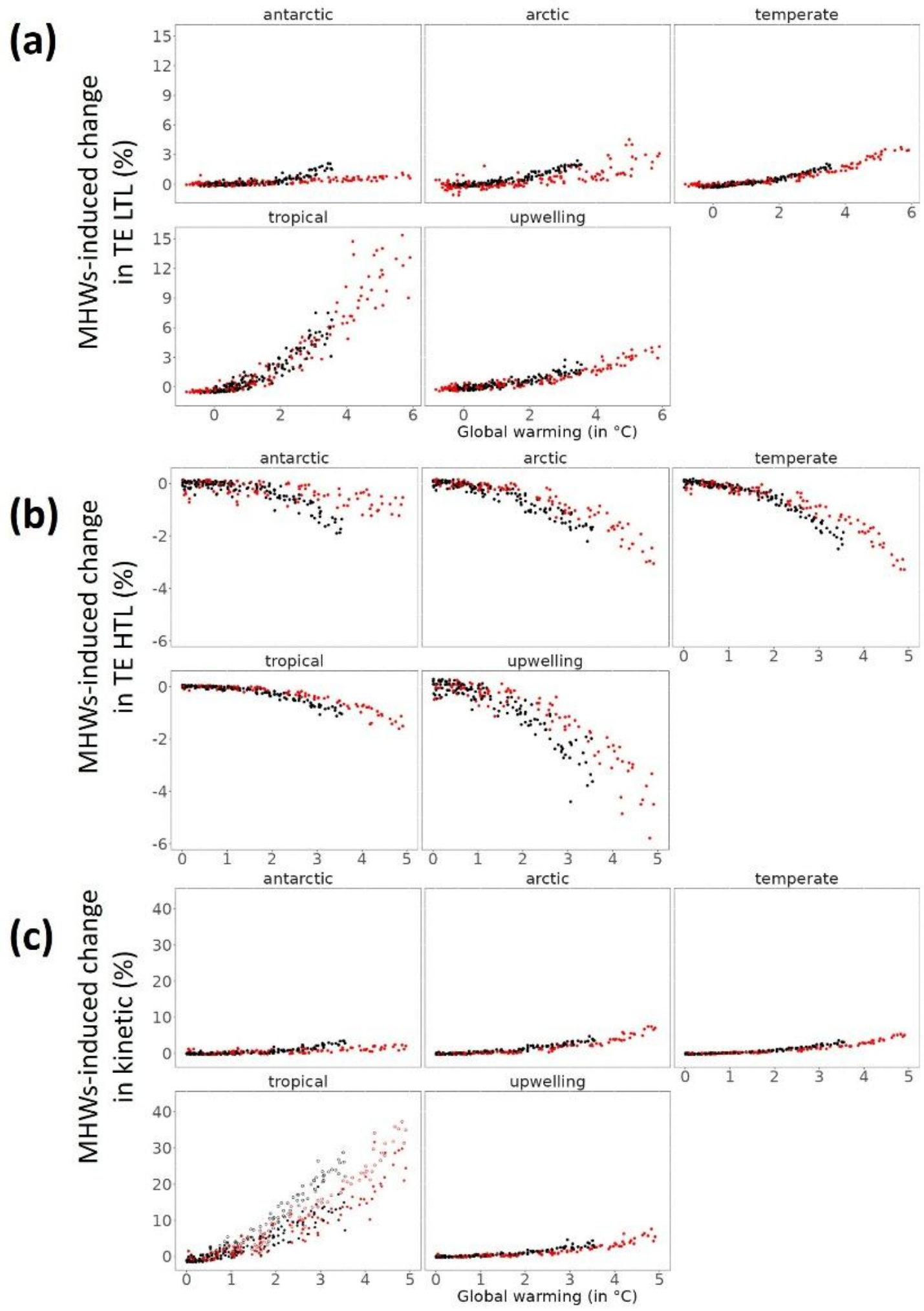

Figure S8: biomes' biomass flow parameters responses to MHWs occurrence under global warming level above preindustrial level (1850-1900) under fixed baseline hypothesis. Panels (a), (b), and (c) represent, for each biome, MHWs-induced effects on TE LTL, TE HTL, and kinetic, respectively. Each point represents a yearly value, with red and black markers corresponding to IPSL and GFDL forcing, respectively.

Appendix S9: NPP and Mesozooplankton

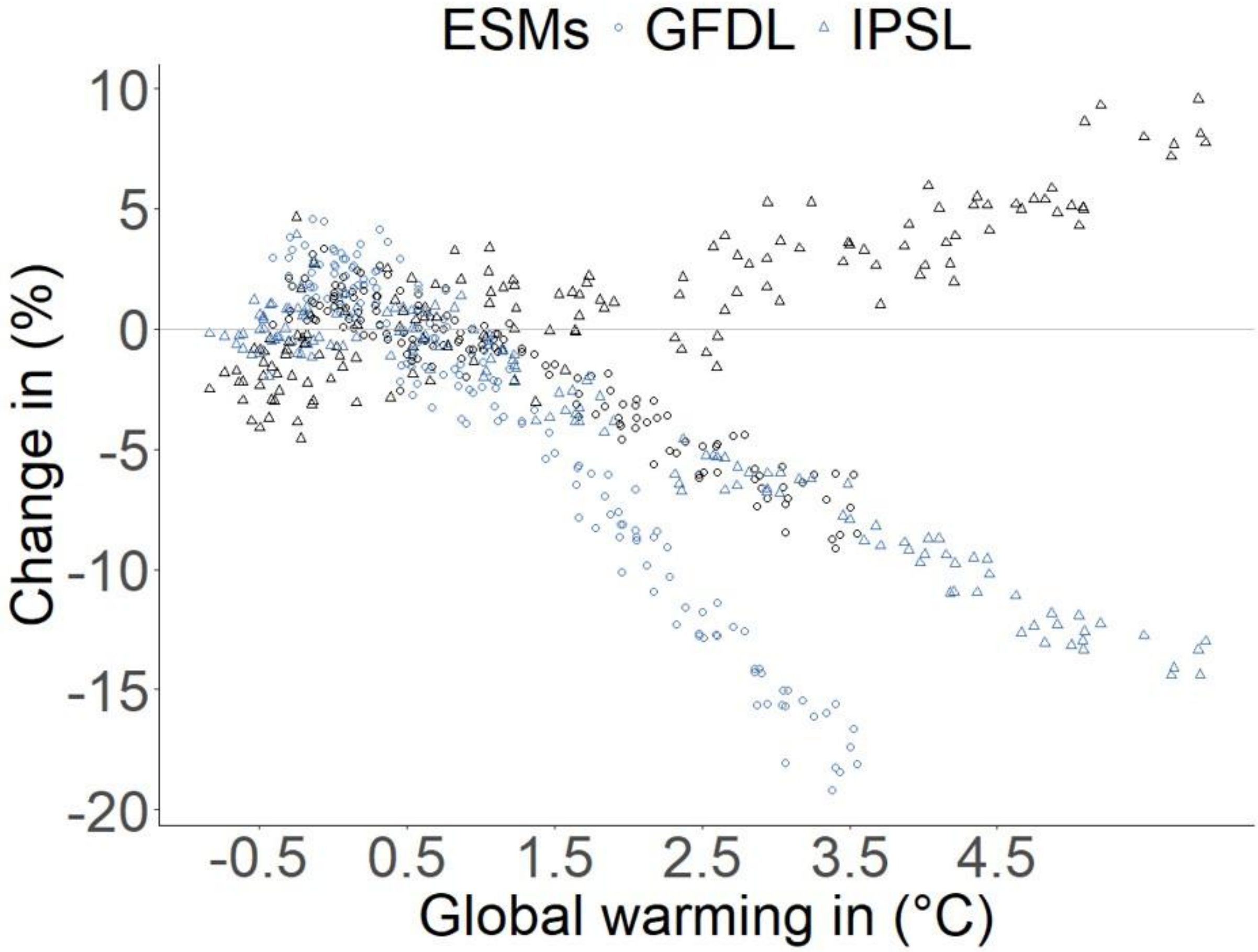

Figure S9: NPP and Mesozooplankton evolution under global warming level above preindustrial level (1850-1900) according to GFDL and IPSL. Each marker represents a yearly value. Blue and black colours correspond to mesozooplankton and NPP, respectively; triangles and circles represent the IPSL and GFDL models, respectively**.**

Appendix S10: Additional biomass loss associated with acclimation scenario to MHWs for both fixed and moving baseline.

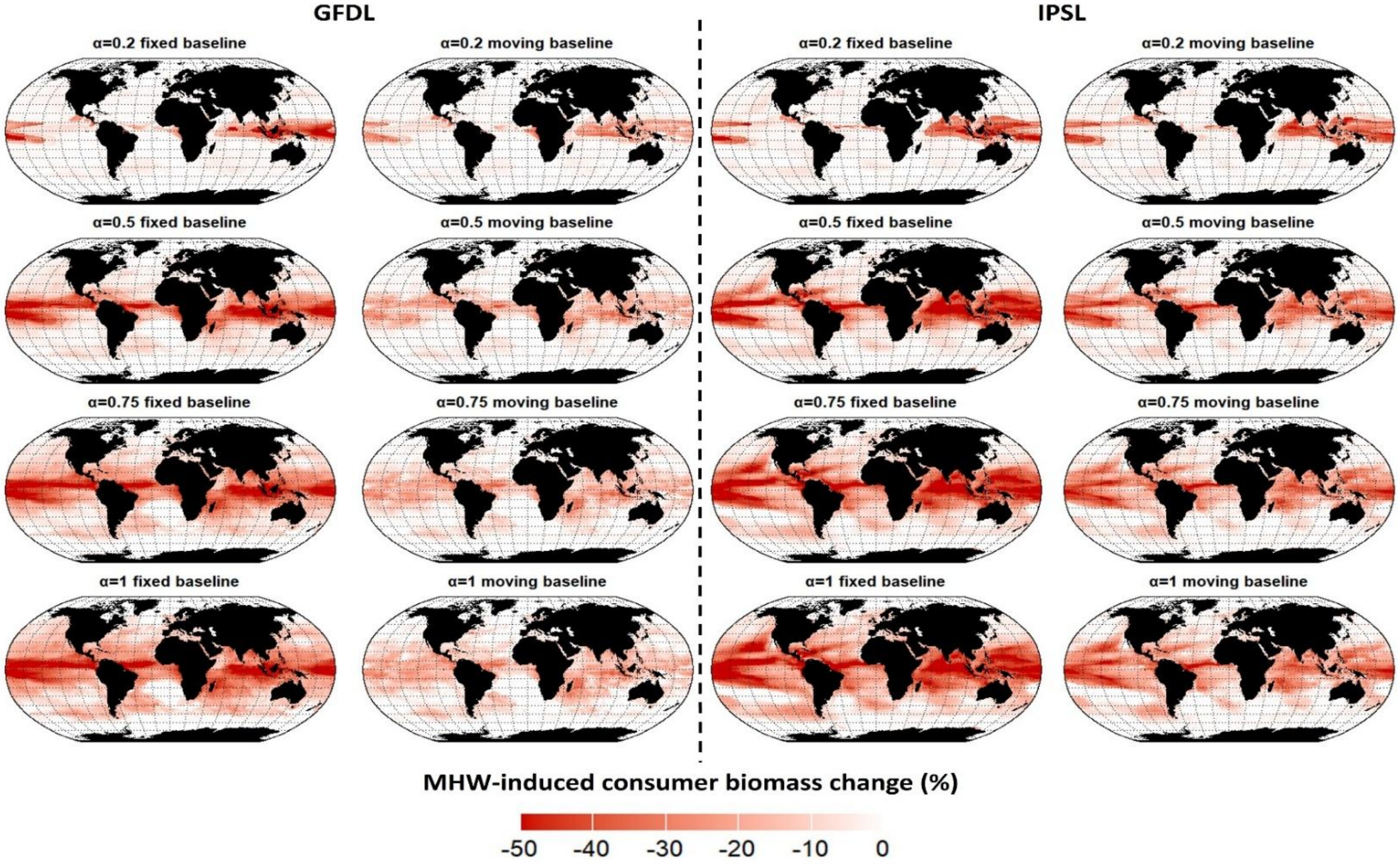

Figure S10: Spatial pattern of MHWs-associated additional biomass loss associated with a 2°C global warming level above preindustrial level (1850-1900). Projections are shown under GFDL (first two columns) and IPSL (last two columns) forcings across different acclimation scenarios. The left and right columns for each model correspond to fixed and moving baselines, respectively. Map lines delineate study areas and do not necessarily depict accepted national boundaries

Appendix S11: Maps of IPSL total consumer biomass change, standard deviation over acclimation scenario to MHWs, and MHWs-associated additional biomass loss associated with a +2°C global sea surface warming level above preindustrial level

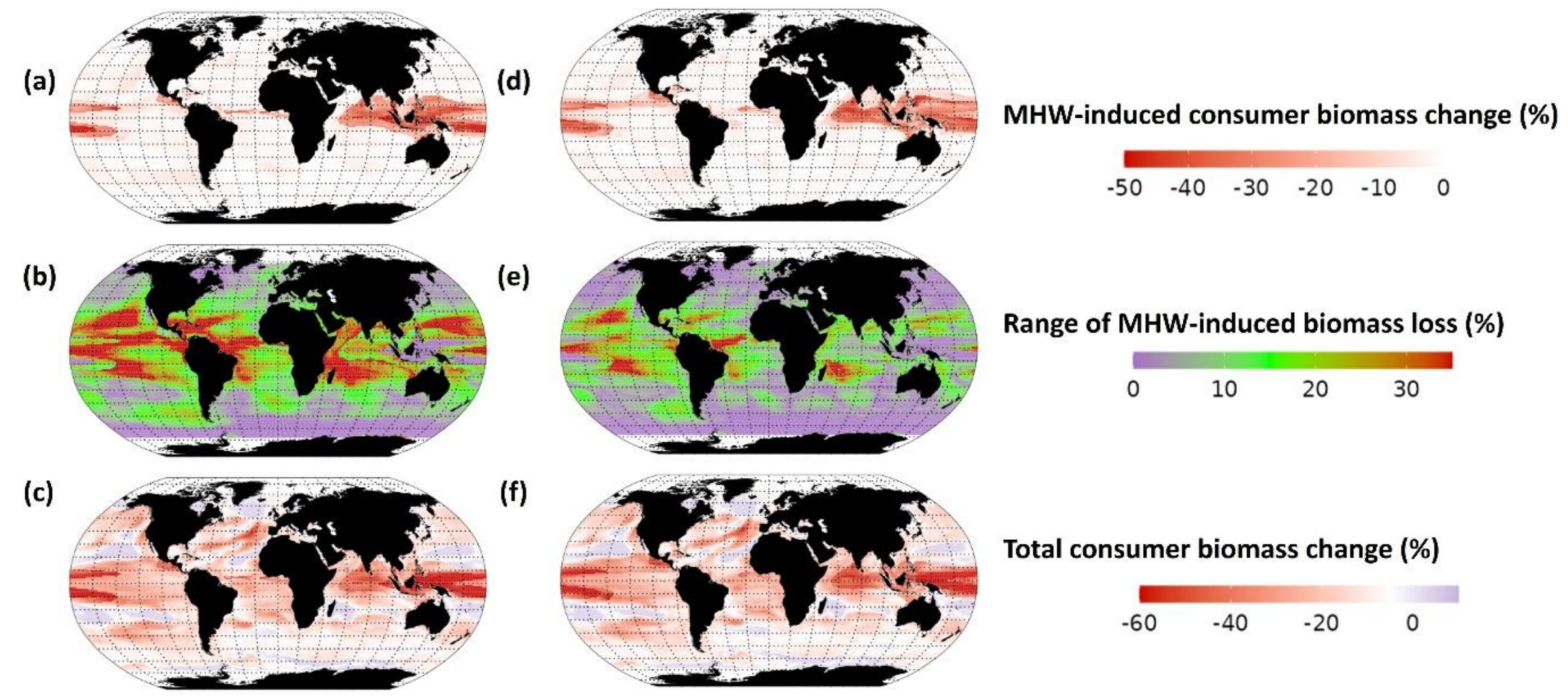

Figure S11: Spatial pattern of projected change in consumer biomass under a +2°C global warming level above preindustrial levels, under IPSL forcing. (a) and (d) additional loss of consumer biomass associated with MHWs over the loss associated with long-term warming under α=0.2 scenario. (b) and (e) range of additional loss of consumer biomass associated with MHWs according to α scenarios (from α = 0 to 1). (c) and (f) spatial pattern of total consumer biomass change relative to the average across the reference period of 1995-2014 under α=0.2 scenario. Panels (a), (b), and (c) correspond to a fixed baseline, while panels (d), (e), and (f) correspond to a moving baseline. Map lines delineate study areas and do not necessarily depict accepted national boundaries

Appendix S12: NPP and mesozooplankton map associated with a 2°C global sea surface warming level above preindustrial level (1850-1900) under GFDL and IPSL

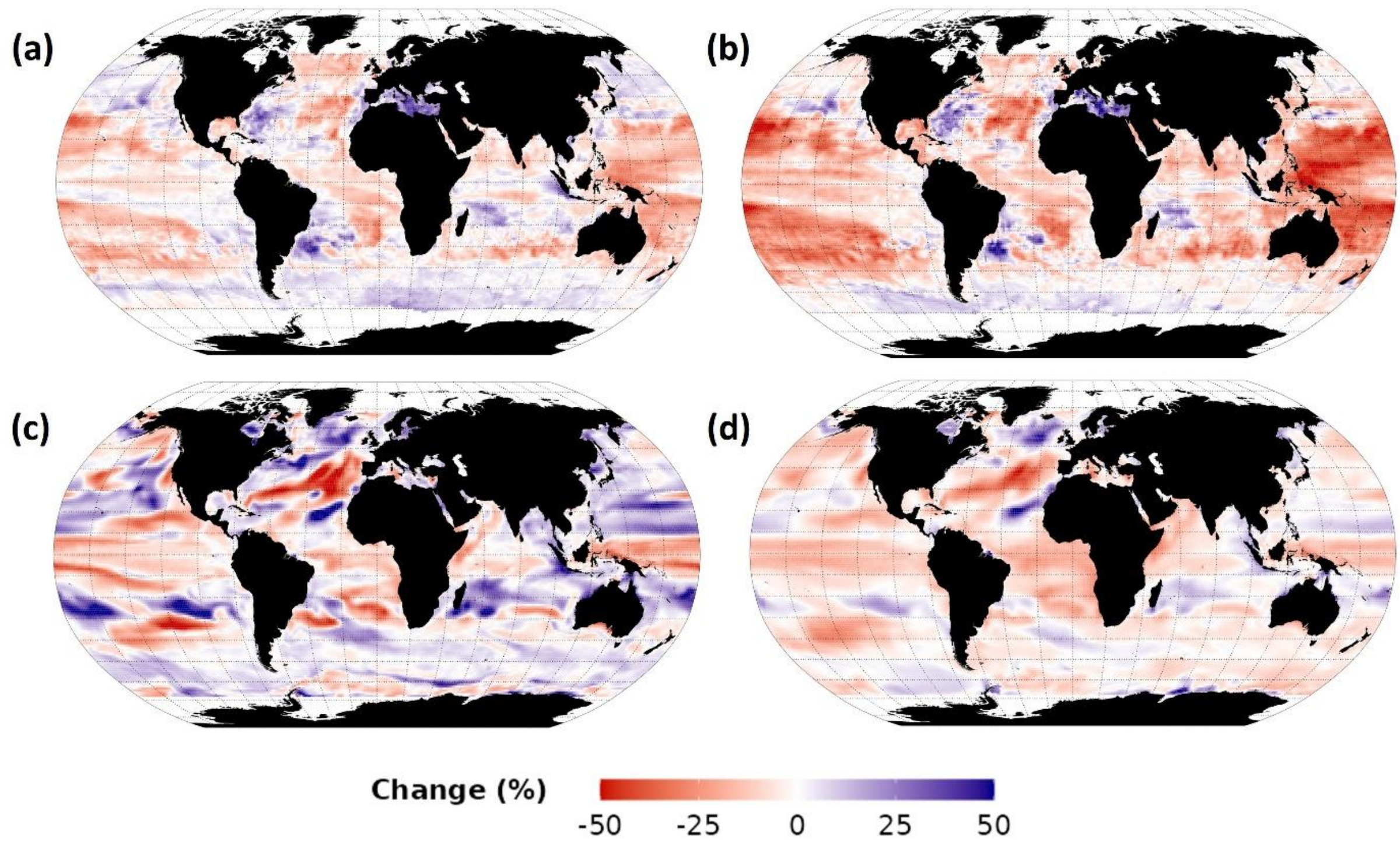

Figure S12: Spatial pattern of NPP and mesozooplankton associated with a 2°C global sea surface warming level above preindustrial level (1850-1900) under GFDL and IPSL forcings. (a) and (b) correspond to NPP and mesozooplankton spatial trends under GFDL forcing, while (c) and (d) correspond to NPP and mesozooplankton spatial trends under IPSL forcing. Map lines delineate study areas and do not necessarily depict accepted national boundaries

Appendix S13: Projected changes in trophic group biomass per biome relative to the average across the reference period of 1995-2014 expressed as a function of global warming level above preindustrial levels according to GFDL forcing, α=0.2, and moving baseline

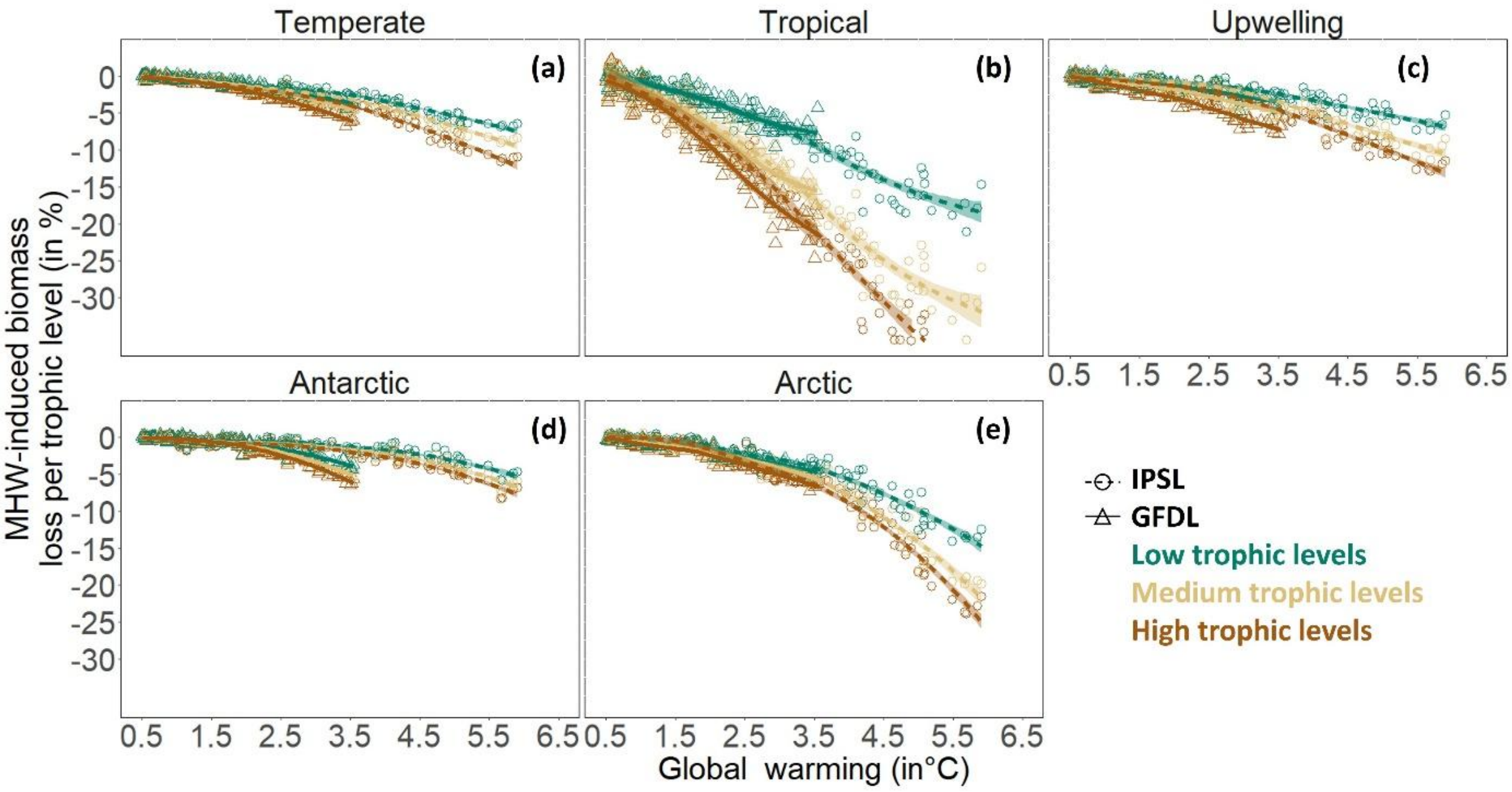

Figure S13: Projected changes in trophic group biomass per biome relative to the average across the reference period of 1995-2014 expressed as a function of global warming level above preindustrial levels according to GFDL and IPSL forcings, α=0.2, and moving baseline. Panels (a)–(e) correspond to the Temperate, Tropical, Upwelling, Antarctic, and Arctic biomes, respectively. Green, gold, and brown correspond to low, medium, and high trophic levels, respectively. Each marker represents a yearly value.

Appendix S14: EcoTroph-Dyn MHWs' simulation compared to Fish-MIP models

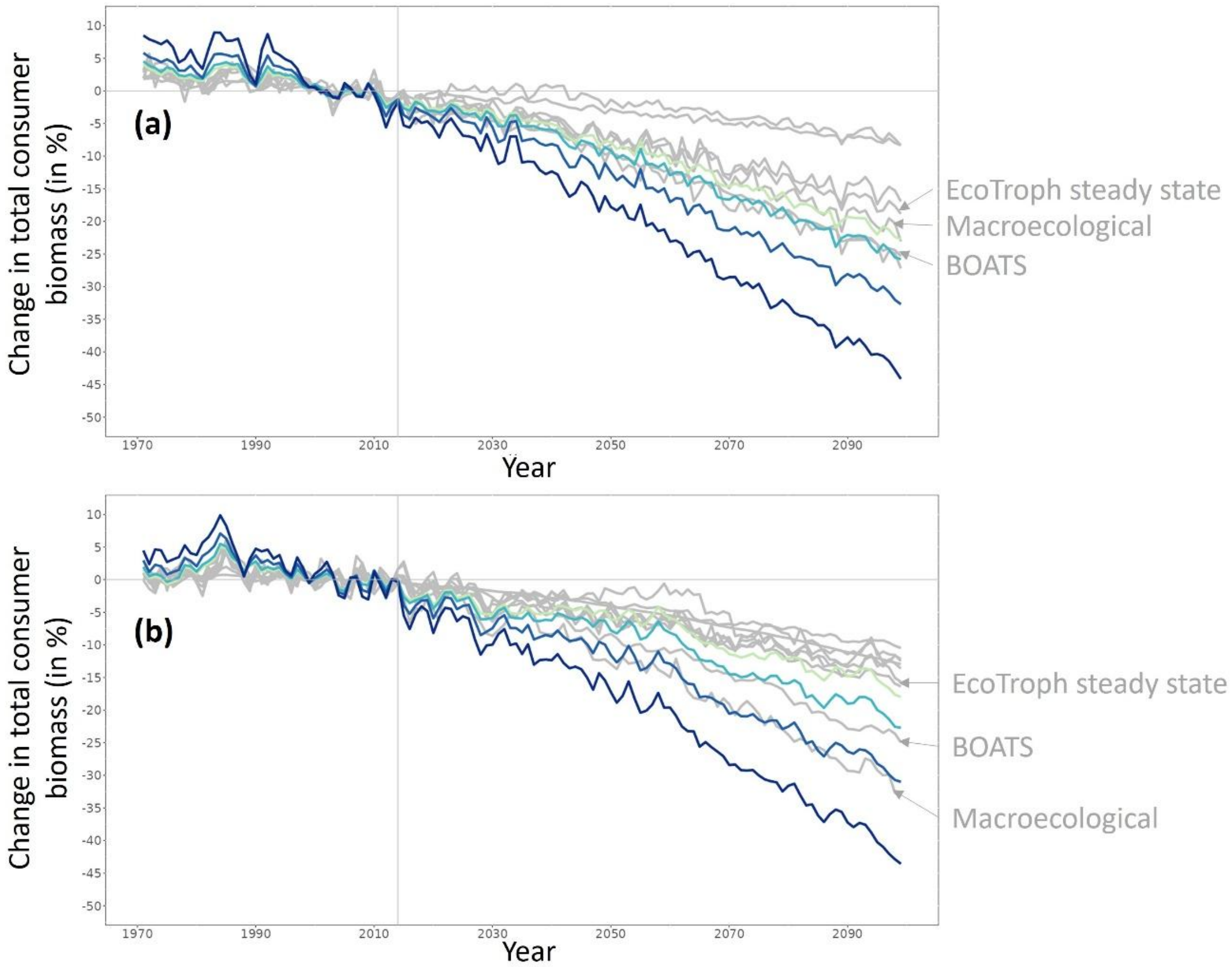

Figure S14: EcoTroph-Dyn and Ensemble projections of total consumer biomass changes, relative to 1995–2014, under SSP5-8.5 (adapted from Guibourd de Luzinais et al 2023). (a) based on GFDL/MEMs combinations; (b) on IPSL/MEMs combinations. All values are relative to the standardised reference period of 1995–2014. The vertical grey line indicates the last year of the historical period. Green, cyan, blue, and dark blue correspond to EcoTroph-Dyn full acclimation (α=0), partial acclimation (α=0.2 and 0.5), and no acclimation (α =1; all species die when they are under thermal stress), projections, respectively.
